# Inhibition of Bcl-2/Bcl-xL and c-MET causes synthetic lethality in model systems of glioblastoma

**DOI:** 10.1038/s41598-018-25802-0

**Published:** 2018-05-09

**Authors:** Yiru Zhang, Chiaki Tsuge Ishida, Chang Shu, Giulio Kleiner, Maria J. Sanchez-Quintero, Elena Bianchetti, Catarina M. Quinzii, Mike-Andrew Westhoff, Georg Karpel-Massler, Markus D. Siegelin

**Affiliations:** 1 Department of Pathology & Cell Biology, Columbia University Medical Center, NY New York, USA; 2 Department of Neurology, Columbia University Medical Center, New York, NY USA; 3 Department of Neurosurgery, Ulm University Medical Center, Ulm, Germany; 4 Department of Pediatrics and Adolescent Medicine, Ulm University Medical Center, Ulm, Germany

## Abstract

Recent data suggest that glioblastomas (GBM) activate the c-MET signaling pathway and display increased levels in anti-apoptotic Bcl-2 family members. Therefore, targeting these two deregulated pathways for therapy might yield synergistic treatment responses. We applied extracellular flux analysis to assess tumor metabolism. We found that combined treatment with ABT263 and Crizotinib synergistically reduces the proliferation of glioblastoma cells, which was dependent on dual inhibition of Bcl-2 and Bcl-xL. The combination treatment led to enhanced apoptosis with loss of mitochondrial membrane potential and activation of caspases. On the molecular level, c-MET-inhibition results in significant energy deprivation with a reduction in oxidative phosphorylation, respiratory capacity and a suppression of intracellular energy production (ATP). In turn, loss of energy levels suppresses protein synthesis, causing a decline in anti-apoptotic Mcl-1 levels. Silencing of Mcl-1 enhanced ABT263 and MET-inhibitor mediated apoptosis, but marginally the combination treatment, indicating that Mcl-1 is the central factor for the induction of cell death induced by the combination treatment. Finally, combined treatment with BH3-mimetics and c-MET inhibitors results in significantly smaller tumors than each treatment alone in a PDX model system of glioblastoma. These results suggest that c-MET inhibition causes a selective vulnerability of GBM cells to Bcl-2/Bcl-xL inhibition.

## Introduction

Malignant glial brain tumors remain to be incurable. Out of this group, the most common primary malignant brain tumor is glioblastoma^1^. Although there have been recent advances in the molecular diagnosis and characterization of these tumors, they still remain therapeutically resistant. In part, this can be linked to heterogeneity, which is exemplified by the simultaneous activation of multiple different pathways that are often times related to kinase signaling.

Concerning kinase pathways, it is well accepted that several of those pathways are activated in GBMs. Most notably, there are alterations in the PI3K signaling pathway caused by common alterations/mutations in the receptor kinase protein, EGFR, or the phosphatase, PTEN. Being not as well recognized as the key PI3K signaling molecules, the c-MET kinase receptor along with its ligand HGF have shown significance in glioblastoma^2,3^. For instance, c-MET appears to be important for the growth and maintenance of stem-like GBM cells, a population of tumor cells within glial brain tumors that is responsible for therapeutic resistance and progression^2,3^.

The anti-apoptotic Bcl-2 family members are a cornerstone in cell death regulation in glioblastoma cells. They can be inhibited by selective compounds, called BH3-mimetics that elicit on-target efficacy in the nanomolar range. Being an elegant and modern approach to tackle tumor cells, BH3-mimetics have been part of several preclinical and clinical drug combination therapies^4^. Our group has recently shown that IDH1 mutated gliomas might be particularly vulnerable to BH3-mimetics, an approach that offers a patient-tailored solution for the treatment of brain tumors.

Tumor cell metabolism is regulated by kinases and oncogenes which tumor cells are addicted to. The classical hallmark is the phenomenon discovered by a german biochemist, Otto Warburg, who found that despite the abundant presence of oxygen tumor cells are more inclined to metabolize glucose via glycolysis to lactate. While at the first glance this appears to be uneconomically it permits tumor cells to entertain anabolic biosynthesis, e.g. serine biosynthesis for one carbon metabolism, nucleotides and so forth. Thus, targeting kinase signaling will interfere with energy production in cancer cells and inevitably exacerbate metabolic vulnerabilities that are therapeutically targetable.

In this report, we provide evidence that targeting c-MET renders GBM cells vulnerable to dual Bcl-2/Bcl-xL inhibition mediated intrinsic apoptosis.

## Results

### Inhibition of c-MET synergizes with Bcl-2/Bcl-xL antagonism

**Figure 1:**
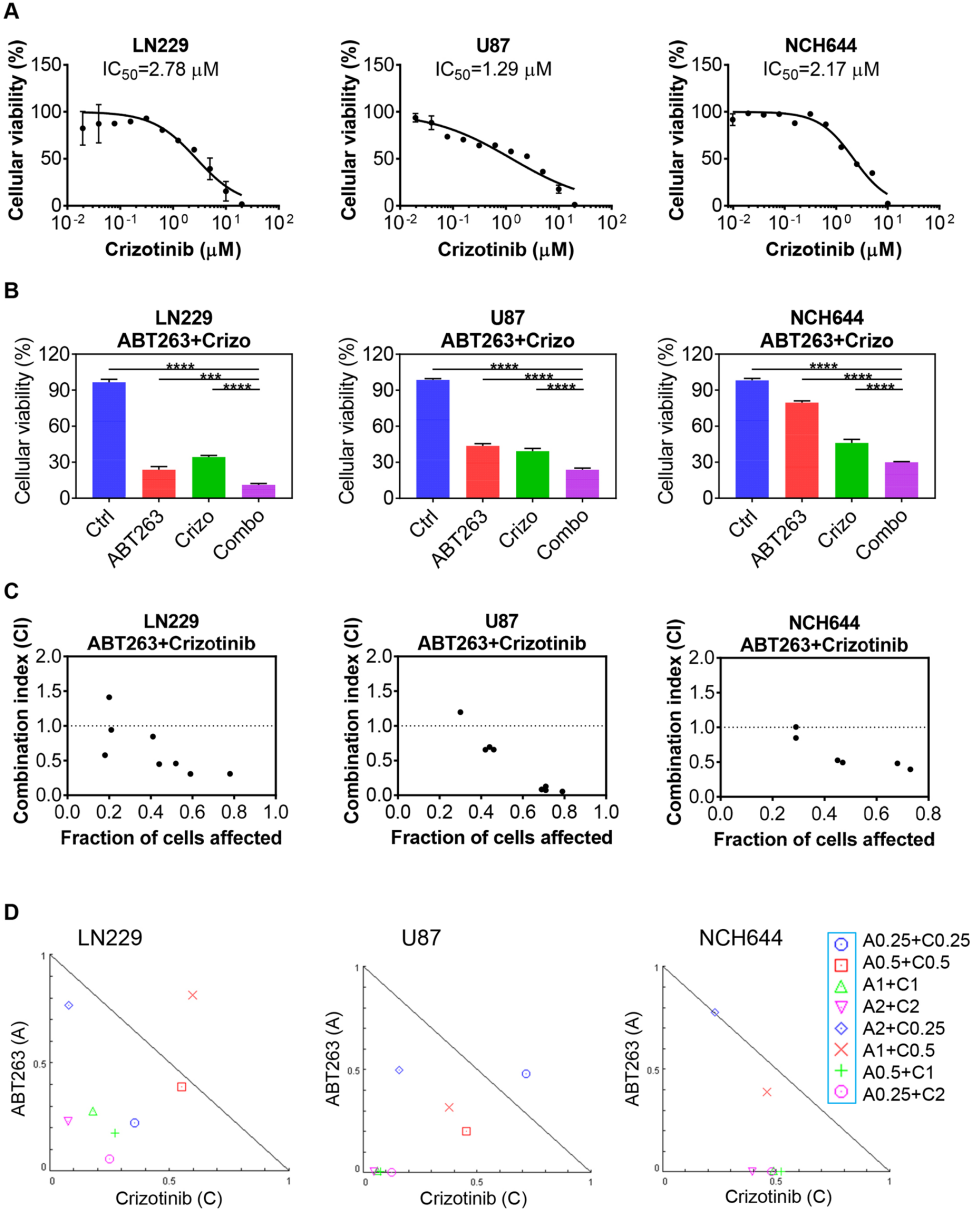
Synergistic interaction of c-MET and Bcl-2/Bcl-xL inhibition in established and stem-like GBM cells. (**A**) LN229, U87 and NCH644 stem-like GBM cells were treated with increasing concentrations with c-MET inhibitor, Crizotinib, and analyzed for cellular viability (3d). Shown are means and SD. Non-linear regression analysis was applied to calculate IC_50_ values. n = 3 (**B**) The same cell lines as in A were treated with suboptimal dosages of ABT263, Crizotinib or the combination and analyzed for cellular viability after 72 h. Shown are means and SD. n = 3 (**C**,**D**) Based on viability assays, CI values were calculated, using the software CompuSyn (ComboSyn, Inc., Paramus, NJ, U.S.A.). CI values below 1 indicate synergistic interactions, whereas values above 1 show antagonism (CI values vs. fraction affected). Isobolograms are further highlighting the synergistic interaction between ABT263 and Crizotinib. The applicable concentrations (in μM) for the used compounds are shown on the right side of the figure. A: ABT263, C: Crizotinib.

First, we validated that Crizotinib acts on-target by confirming in whole cell protein lysates of NCH644 GBM stem-like cells and U87 cells that Crizotinib elicits a reduction in phosphorylation of c-MET (Supplementary Figure 1C), confirming that this compound is active in our model systems. Next, we tested the effects of Crizotinib on cellular viability and subsequently determined IC_50_ values in LN229, U87 and NCH644 cells. All IC_50_ values were found to be in the low micro molar range (Fig. 1A). To test the hypothesis that c-MET inhibition and Bcl-2/Bcl-xL inhibition acts synergistically on the reduction of cellular proliferation in model systems of glioblastoma, NCH644, GBM stem-like cells and U87 and LN229 cells were treated with a range of concentrations of the c-MET inhibitor, Crizotinib, the Bcl-2/Bcl-xL inhibitor, ABT263 or the combination of the two reagents. We found that in all model systems tested, Crizotinib and ABT263 reduced the proliferation of GBM cells in a synergistic manner, revealing CI values of less than 1.0 (Fig. 1B–D). To better appreciate the impact of ABT263 on Crizotinib mediated efficacy, we tested dose escalation of the c-MET inhibitor in the presence or absence of ABT263. As anticipated, we found a reduction in the IC_50_ values of Crizotinib in the presence of ABT263 in all model systems tested (Supplementary Figure 1A,B).

Given the presence of several anti-apoptotic Bcl-2 family members that can have an impact on cell death signaling, such as Bcl-2, Bcl-xL and Mcl-1, and the fact that for these proteins selective BH3-mimetics (inhibitors) are available, we assessed the impact of each individual Bcl-2 family member on the effect of Crizotinib mediated reduction of cellular viability (Fig. 1B and supplementary Figure 2). To this purpose, NCH644, U87 and T98G GBM cells were treated with selective BH3-mimetics, A1210477 (Mcl-1 inhibitor), WEHI-539 (Bcl-xL inhibitor), ABT199 (Bcl-2 inhibitor) in the presence or absence of Crizotinib. Our findings highlight that Crizotinib mediated reduction in cellular viability was most pronounced when both Bcl-2 and Bcl-xL were inhibited (ABT263).

**Figure 2:**
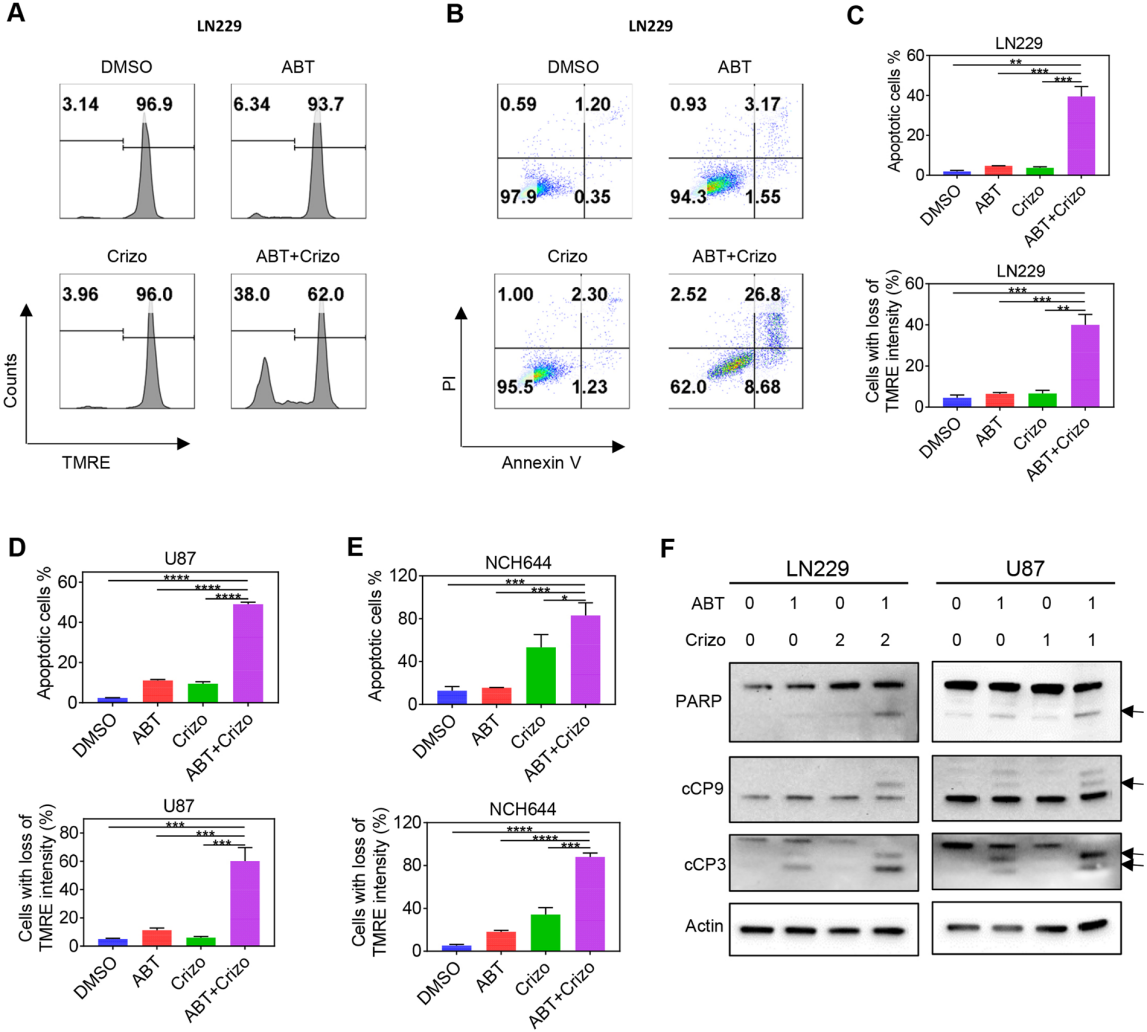
Enhanced apoptosis induction and dissipation of mitochondrial membrane potential by c-MET and Bcl-2/Bcl-xL inhibition. (**A)** LN229 GBM cells were treated with ABT263 (ABT), Crizotinib (Crizo) or the combination of both and analyzed for mitochondrial membrane potential by TMRE staining with subsequent flow cytometry. (**B**) LN229 GBM cells were treated as in A, stained with Annexin V/propidium iodide and analyzed by multi-parametric flow cytometry. (**C–E**) Quantifications of Annexin and TMRE staining flow cytometric results in LN229, U87 and stem-like GBM cells. Shown are means and SD. n = 3. (**F**) LN229 and U87 GBM cells were treated with ABT263, Crizotinib (Crizo) and the combination treatment (1d). Whole cell protein lysates were prepared and analyzed by conventional Western blotting for the expression of PARP, cCP9 (cleaved caspase 9), cCP3 (cleaved Caspase-3). Actin serves as loading control. Arrows highlight the cleavage fragments of the respective proteins. Concentrations are in μM.

To gain a better understanding of the cell death mechanisms involved in the combination treatment of ABT263/Crizotinib, we conducted Annexin V/propidium iodide staining, followed by multi-parametric flow cytometry. While vehicle or single treatments displayed little impact on apoptosis induction, the combination treatment resulted in a significant increase in Annexin V positive cells, suggesting that the synergistic effect of the combination treatment is largely mediated by enhanced activation of apoptosis (Fig. 2B–D and Supplementary Figure 2). Supporting the findings above, cells treated with the combination treatment of ABT263 and Crizotinib showed a more significant decrease in loss of mitochondrial membrane potential as assessed by TMRE staining (Fig. 2A,C–E). Apoptosis is often accompanied by caspase activation. Therefore, we assessed our various treatments (ABT263, Crizotinib and the combination) for caspase cleavage by conventional western blotting. The results indicate that the combination treatment led to an enhanced cleavage of caspases and PARP (Fig. 2F). These results are in keeping with the findings observed in the Annexin V assay and TMRE staining.

### Interference with c-MET signaling impacts Mcl-1 protein levels and the Noxa-Mcl-1 ratio

**Figure 3:**
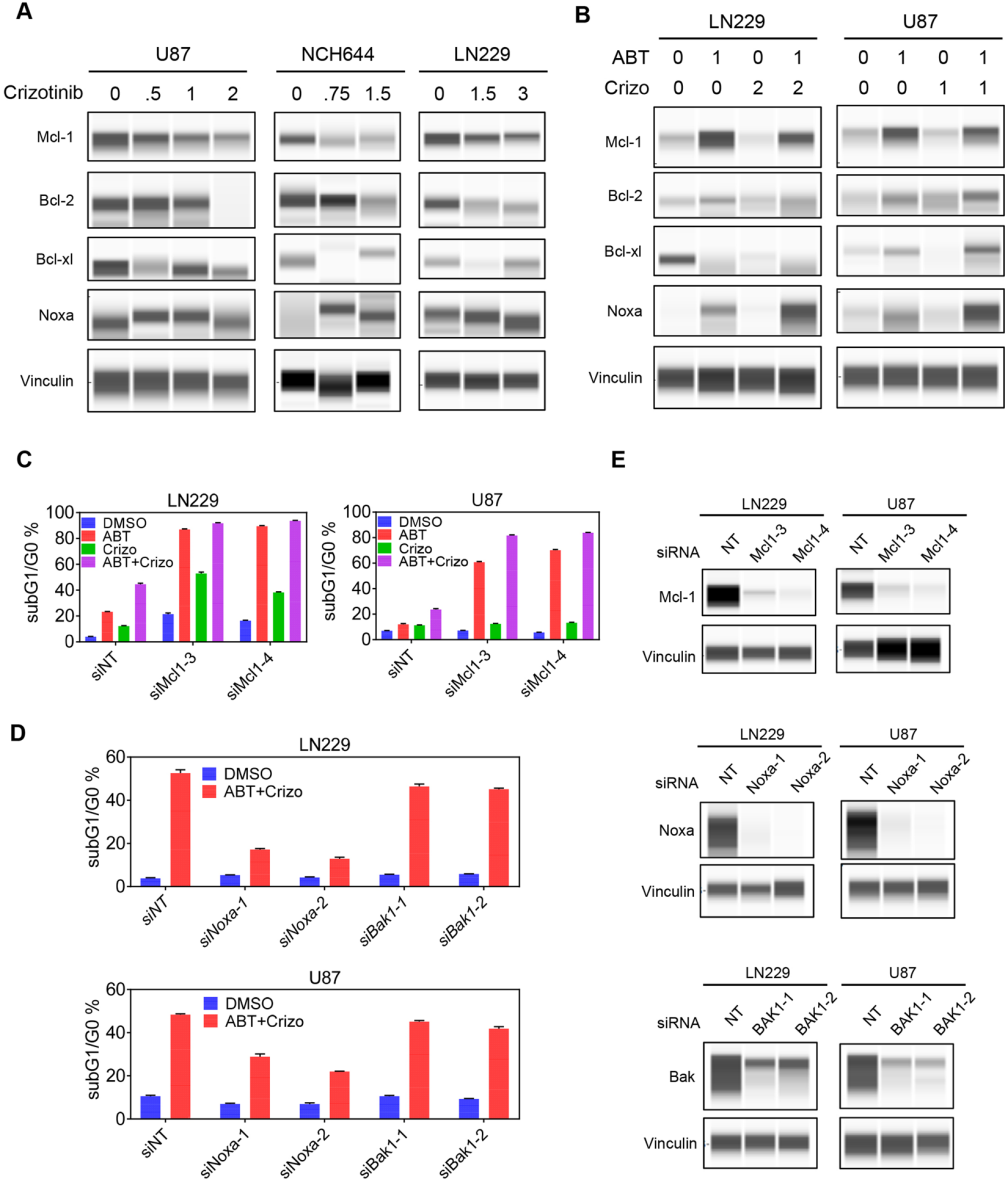
Protein levels of anti- and pro-apoptotic Bcl-2 family members upon treatment with c-MET inhibitor, ABT263 or the combination of both. (**A)** U87, NCH644 and LN229 GBM cells were treated with increasing concentrations of Crizotinib (in μM) for 1d. Thereafter, whole cell lysates were prepared and analyzed by capillary electrophoresis on the Wes Instrument (Proteinsimple Inc.) for the expression of Mcl-1, Bcl-2, Bcl-xL, Noxa and Vinculin. (**B)** LN229 and U87 GBM cells were treated with ABT263 (ABT), Crizo (Crizotinib) or the combination for 1 d (concentrations in μM). Whole cell protein lysates were prepared and analyzed for the same markers as in A. (**C**) LN229 and U87 GBM cells were transfected with two siRNAs targeting Mcl-1. Thereafter, cells were treated with either ABT263, Crizotinib or the combination (1d). And then harvested, fixed, stained with propidium iodide and analyzed by flow cytometry. Shown are means and SD. (**D)** LN229 and U87 GBM cells were transfected with Noxa or Bak specific siRNAs and treated with the combination of 1 μM ABT263 (ABT) and 2 μM Crizotinib (Crizo) for 1d. Cells were processed and analyzed as in C. Shown are means and SD. (**E)** LN229 and U87 GBM cells were transfected with Bak, Noxa or Mcl-1 specific siRNAs. Thereafter, whole protein lysates were prepared and analyzed for the expression of Noxa, Bak and Mcl-1 by capillary electrophoresis on the Wes Instrument (Proteinsimple, Inc.). Concentrations are in μM.

Next, we asked the question by which underlying molecular mechanism the proposed combination treatment of ABT263 and Crizotinib elicits its enhanced pro-apoptotic effects. To this purpose, we assessed the protein levels of Mcl-1, Bcl-2, Bcl-xL and Noxa after different treatment conditions in U87, LN229 and NCH644 stem-like GBM cells. To account for the impact of c-MET inhibition on these Bcl-2 family proteins, U87, LN229 and NCH644 cells were treated with dose escalations of c-MET inhibitor, Crizotinib, for 24 h. Thereafter, whole cell protein lysates were collected and analyzed by capillary electrophoresis (Fig. 3A). Most relevantly to the observed synergism between c-MET and Bcl-2/Bcl-xL inhibition, we appreciated a dose-dependent down-regulation of the anti-apoptotic Mcl-1 protein (Fig. 3A). Consistently, the literature indicates a pivotal role for Mcl-1 as a mediator of resistance for Bcl-2/Bcl-xL inhibition mediated cell death (see discussion for more details and references). Despite the changes in Mcl-1 protein levels upon treatment with Crizotinib, we found a dose-dependent decrease in anti-apopotic Bcl-2 and Bcl-xL as well, further lowering the threshold for intrinsic apoptosis induction (Fig. 3A).

We determined the impact of the combination treatment of Crizotinib and ABT263 on the levels of the Bcl-2 family proteins since ABT263 has been shown to regulate the expression levels of the Bcl-2 family members. While ABT263 usually does not affect Bcl-2 and Bcl-xL, Mcl-1 levels have been reported to be altered by ABT737 and ABT263, respectively (see discussion for references and more details). In agreement with earlier findings, ABT263 elicits an increase in the levels of Mcl-1, which in turn dampens its efficacy on apoptosis induction (Fig. 3B). Counteracting this anti-apoptotic response, inhibition of c-MET by Crizotinib attenuates Mcl-1 mediated increase by ABT263 and further enhances up-regulation of pro-apoptotic Noxa (Fig. 3B), known to intrinsically inhibit Mcl-1 function by promoting the dissociation of BAK from Mcl-1^5–11^. The combination treatment led to a transitory increase of Bcl-2/Bcl-xL, which in turn can be antagonized by ABT263 to further drive intrinsic apoptosis induction.

### Mcl-1 and Noxa are functionally implicated in the combination treatment of ABT263 and Crizotinib

Given the regulation of Mcl-1 protein by c-MET inhibition, we determined the role of Mcl-1 in the various treatments. To this purpose, we utilized two Mcl-1 specific siRNAs and transfected U87 and LN229 GBM cells (Fig. 3C,E). Knockdown efficiency was confirmed by capillary electrophoresis accordingly (Fig. 3E). After transfection, LN229 and U87 GBM cells were treated with vehicle, ABT263, Crizotinib or the combination of both. In agreement with earlier findings from others and our own group, we found that knockdown of Mcl-1 drastically sensitizes for Bcl-2/Bcl-xL mediated cell death (Fig. 3C). To account for the role of Mcl-1 in the combination treatment, we compared the amount of apoptosis induction of cells treated with ABT263 and the combination treatment of ABT263 and Crizotinib in the context of silenced Mcl-1 protein levels (Fig. 3C). Our results suggest that silencing of Mcl-1 does not substantially further enhance DNA-fragmentation in GBM cells that were exposed to ABT263/Crizotinib, suggesting that the effect of the combination treatment might be predominantly mediated by its effect on Mcl-1 and its associated intrinsic antagonist, Noxa (Fig. 3C–D). Further, we evaluated the effect of Mcl-1 silencing on Crizotinib mediated DNA-fragmentation and found that knockdown of Mcl-1 enhances Crizotinib mediated apoptosis (Fig. 3C).

As outlined earlier, Mcl-1 is regulated by an intrinsic pro-apoptotic Bcl-2 family member, Noxa. High levels of Noxa favor sensitivity to Bcl-2/Bcl-xL/Bcl-w targeting BH3-mimetics^8^. Given our observation that especially the combination treatment drives up regulation of Noxa^8,11^, it was tempting to determine the impact of Noxa on ABT263/Crizotinib mediated cell death. To this end, we silenced the expression of Noxa by using two Noxa specific oligonucleotides in U87 and LN229 GBM cells. Capillary protein electrophoresis confirmed the efficacy of the two Noxa related siRNAs (Fig. 3E). As anticipated, siRNA mediated silencing of Noxa attenuates apoptosis induction by ABT263/Crizotinib in U87 and LN229 GBM cells, establishing a key role of Noxa in this drug combination therapy (Fig. 3D). To address the role of BAK in ABT263/Crizotinib mediated apoptosis, we silenced the expression of BAK by using two different siRNAs. Knockdown efficiency was confirmed by capillary electrophoresis in LN229 and U87 GBM cells (Fig. 3E). We found that BAK knockdown was marginally protective after the combination treatment (Fig. 3D).

### Dual suppression of c-Met signaling and Bcl-2/Bcl-xL impairs tumor cell metabolism with a decline in energy levels

**Figure 4:**
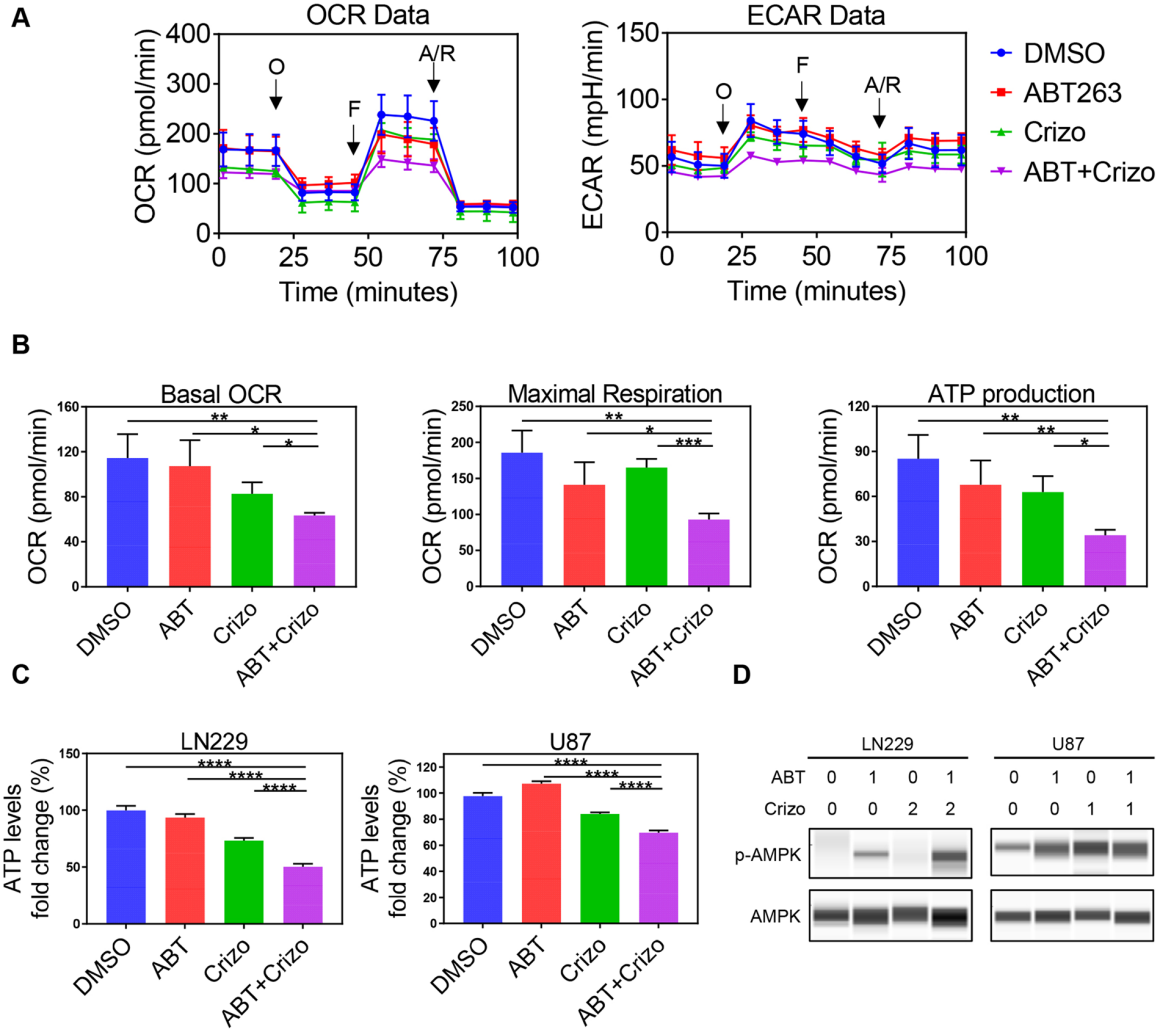
c-MET inhibition reprograms glioblastoma energy metabolism by suppression of oxidative phosphorylation. (**A)** LN229 GBM cells were treated with DMSO, ABT263, Crizotinib (Crizo) or the combination of both and analyzed by extracellular flux analysis on a Seahorse XF24 device (Agilent, Inc.) in the context of a mitochondrial stress test. This assay records the base line oxygen consumption rate of cells (Basal OCR), followed by the injection of Oligomycin (O), the uncoupler (FCCP) and Antimycin/Rotenone (A/R). In addition, we recorded extracellular acidification rate (ECAR) simultaneously along with OCR. Shown are mean values with SD. (**B)** Calculation of individual OXPHOS related parameters are displayed. Shown are mean values with SD. (**C**) LN229 and U87 GBM cells were treated with ABT263, Crizotinib or the combination as indicated. Thereafter, ATP levels were assessed in comparison to the control treated cells. Shown are means and SD. ABT: ABT263, Crizo: Crizotinib. (**D)** LN229 and U87 GBM cells were treated with ABT263, Crizotinib or the combination for 1d. Thereafter, whole cell protein lysates were prepared and analyzed for the expression of p-AMPK (threonine 172) or AMPK by capillary electrophoresis. Concentrations are in μM.

To provide an explanation for the above-observed molecular changes on Bcl-2 family proteins, we hypothesized that these changes might be in part mediated by alterations in tumor cell metabolism upon treatment with the compounds. To this end, we performed extracellular flux analysis in conjunction with a mitochondrial stress test (Fig. 4A). This assay evaluates the baseline mitochondrial oxygen consumption rate, followed by acute administration of a series of OXPHOS modulating compounds. While ABT263 had little impact on mitochondrial oxygen consumption rate, this effect was more pronounced in GBM cells treated with Crizotinib, indicating that c-MET inhibition likely has a direct impact on cellular respiration in human GBM cells (Fig. 4A). Akin to the basal oxygen consumption rate, OXPHOS driven ATP production as well as the maximal respiration rate was reduced in Crizotinib treated GBM cells (Fig. 4B). Despite the suppression of oxygen consumption rate, we did not detect a compensatory increase in extracellular acidification rate (an indicator of glycolytic activity), a phenomenon that is often observed with compounds which suppress OXPHOS (Fig. 4A). To appreciate the impact of ABT263 on the Crizotinib mediated regulation of tumor cell metabolism, we evaluated the combination treatment of ABT263 and Crizotinib in the context of extracellular flux analysis. In keeping with the expectation, the combination treatment synergistically reduced OXPHOS related parameters, explaining in part the effect of the combination treatment on cellular viability (Fig. 4A,B). Regarding extracellular acidification rate (ECAR), we detected a significant decrease in glycolysis upon treatment with ABT263 combined with Crizotinib at baseline and a further pronounced decrease upon stimulation with oligomycin (an inhibitor of complex V of the respiratory chain, which in turn stimulates glycolysis) (Fig. 4A).

To determine the overall impact of metabolism on energy delivering nucleotides, we assessed the levels of ATP in vehicle, ABT263, Crizotinib and ABT263 + Crizotinib treated GBM cells (Fig. 4C). Consistent with the findings from the extracellular flux analysis, the combination treatment reduced ATP levels most significantly (Fig. 4C). In keeping with the total levels of ATP are our findings related to the phosphorylation status of AMPK (threonine 172). While ABT263 treated cells revealed only a settle increase in phosphorylated AMPK, the combination treatment showed a more significant increase, suggesting a state of energy deprivation (Fig. 4D).

### Energy decline leads to suppression of protein synthesis and an activation of endoplasmic reticulum (ER)-stress signaling

**Figure 5:**
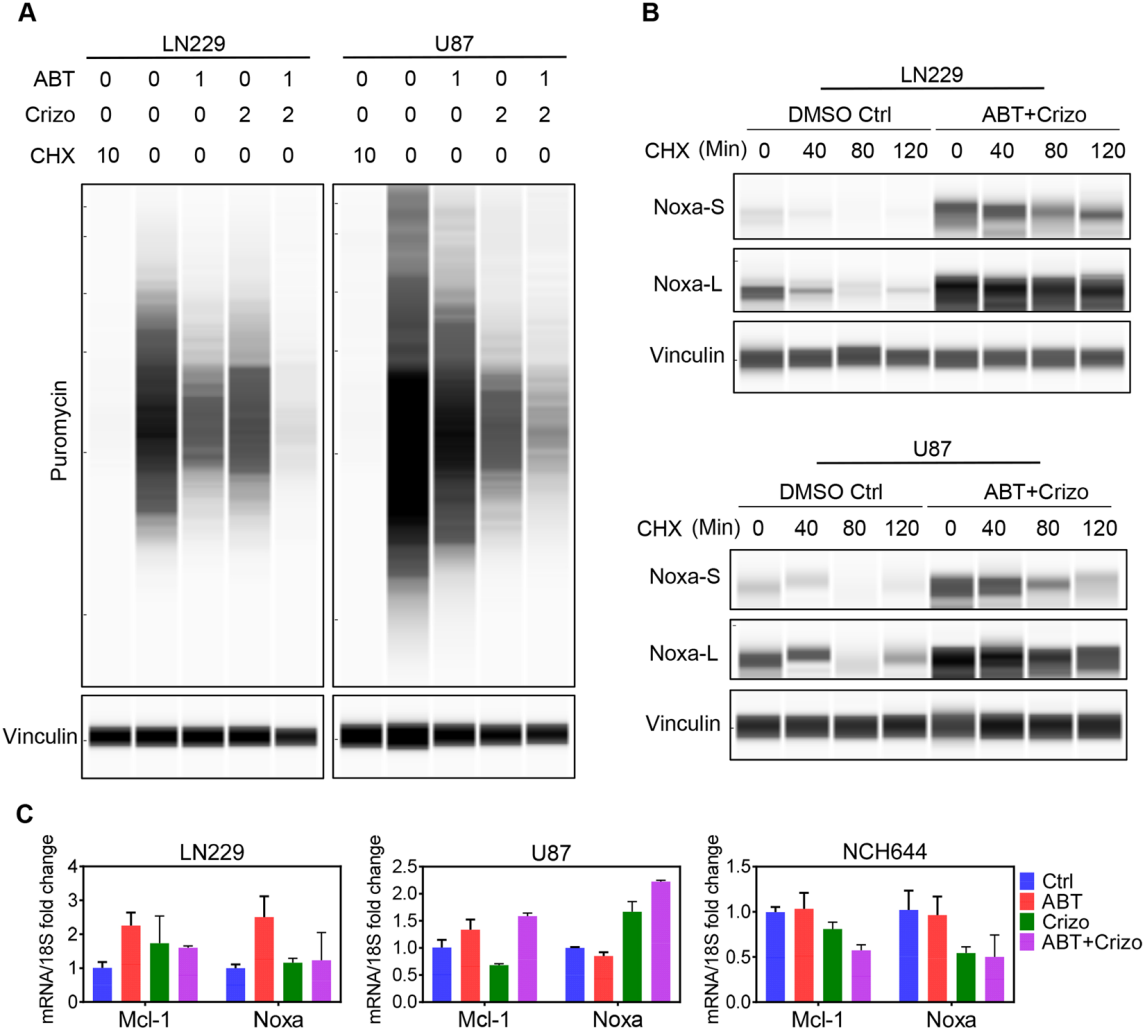
C-MET and Bcl-2/Bcl-xL inhibition suppresses protein synthesis and stabilize pro-apoptotic Noxa. (**A**) LN229 and U87 GBM cells were treated with ABT263, Crizotinib or the combination of both (24 h) (concentrations in μM). At the end of the treatment period, cells were incubated for 2 h with puromycin. Thereafter, whole cell protein lysates were harvested and analyzed by capillary electrophoresis for the expression of puromycin (protein synthesis activity in cancer cells after various treatments referred to as SUNSET assay). Cycloheximide (CHX) serves as a positive control (concentrations are in μg/ml) ABT: ABT263, Criz: Crizotinib. (**B)** LN229 or U87 GBM cells were exposed to cycloheximide in the presence or absence of the combination treatment of ABT263 and Crizotinib. Thereafter, whole protein lysates were isolated and analyzed by capillary electrophoresis. ABT + Crizo: ABT263 1 μM + Crizotinib 2 μM. Noxa-S and Noxa-L refer to the exposure times. L: long exposure; S:short exposure. (**C**) LN229, U87 and stem like GBM cells, NCH644, were treated as indicated for 24 h. RT-PCR was performed for the expression of Mcl-1 and Noxa. Ctrl: Control, ABT: 1 μM ABT263, Crizo: 2 μM Crizotinib.

It is well accepted that depletion of energy leads to a suppression of protein synthesis^12^, thus affecting the levels of proteins that have a short half-life, such as Mcl-1. To test the hypothesis that single and combination treatments affect global protein synthesis, we utilized the SUNSET assay^13^, allowing determining protein synthesis in a non-radioactive manner. In alignment with the extracellular flux analysis as well as with overall ATP levels, we found that Crizotinib and the combination treatment of ABT263 and Crizotinib suppressed protein synthesis in LN229 and U87 GBM cells (Fig. 5A).

Since energy depletion may lead to endoplasmic reticulum stress induction, we evaluated the protein levels of markers indicative of ER-stress. We found that the combination treatment elevated protein levels of GRP78, ATF4 and ATF3, which are classical markers of ER-stress induction (Supplementary Figure 4).

To have a better understanding in the regulatory requirements of Noxa and Mcl-1, we determined their mRNA levels as well as the protein stability for Noxa after treatment with ABT263, Crizotinib or the combination of both. While in LN229 we found an increase in Mcl-1 mRNA levels, we observed a slight decrease in U87 and NCH644 cells (Fig. 5B,C). With the exception of NCH644, the combination treatment led to an elevation of Mcl-1 mRNA levels, in keeping with an induction of a stress-response. Concerning Noxa mRNA levels, we detected a slight increase in LN229 and a more pronounced induction in U87 cells, but a decrease in NCH644 stem like cells upon treatment with Crizotinib (Fig. 5C). The combination treatment of ABT263/Crizotinib increased Noxa mRNA levels in U87 cells, but decreased it in NCH644 cells (Fig. 5C). However on protein levels, we found an upregulation of Noxa levels upon administration of the combination treatment (see above), suggesting that non-transcriptional events are implicated to enhance Noxa protein levels. Given our cell line dependent findings related to Noxa mRNA levels upon treatment with the combination treatment of ABT263/Crizotinib, we tested the hypothesis that ABT263/Crizotinib might enhance the stability of Noxa. To this purpose, we conducted a cycloheximide block experiment in the presence or absence of ABT263/Crizotinib in LN229 and U87 GBM cells. In keeping with our hypothesis, we detected enhanced stability of Noxa in the presence of ABT263/Crizotinib (Fig. 5B).

### The combination treatment of ABT263 and Crizotinib leads to a more significant reduction in tumor growth than vehicle or single treatments in a patient-derived xenograft model of human GBM

*In vivo* efficacy is crucial for the translation of promising therapeutics or strategies into the clinic. Therefore, preclinical animal models are necessary to provide a foundation. While established cell line models giving rise to xenografts provide tractable systems, they are not as representative of human disease as patient-derived xenografts. Therefore, we utilized the patient-derived xenograft model, GBM12, to test the hypothesis that the combination treatment of ABT263 and Crizotinib leads smaller tumor than vehicle or each compound on its own.

**Figure 6:**
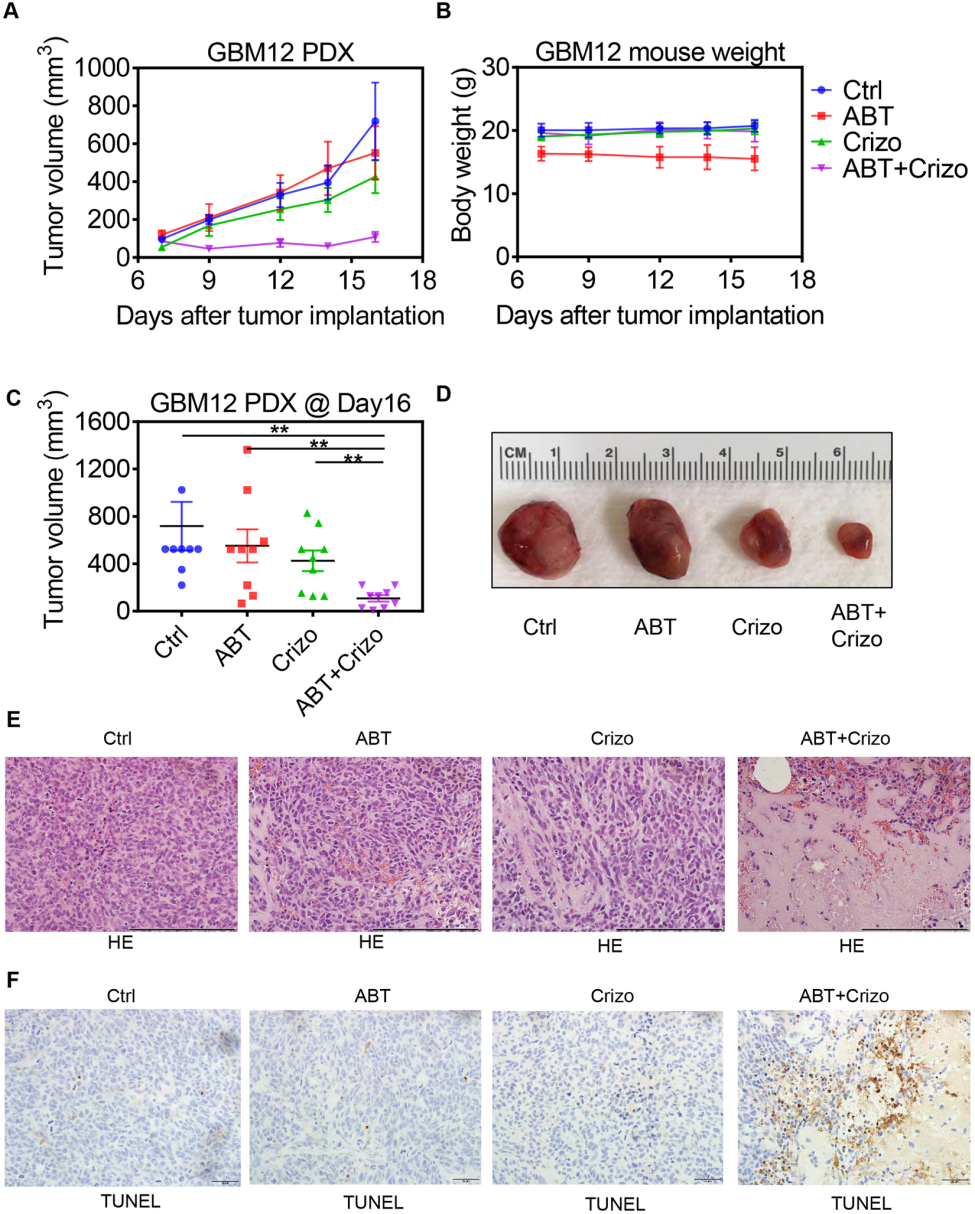
Dual inhibition of c-MET and Bcl-2/Bcl-xL leads to enhanced tumor growth suppression in a PDX model of GBM. (**A**) GBM12 PDX cells were implanted into the subcutis of immunocompromised Nu/Nu mice. After tumors were established (palpable), randomization was performed to assign four distinct treatment groups. These groups are designated as: Control; ABT263 (ABT) (75 mg/kg); Crizotinib (Crizo) (50 mg/kg) and ABT263 + Crizotinib (ABT + Crizo). Shown are tumor volumes over time. Values are means and SD. n = 9 in each group. (**B**) The indicated groups were assessed for body weight. Shown are means and SD. (**C**) Shown is the last measurement as a dot-plot (means and SD) with corresponding statistical analysis. n = 9. (**D)** Gross images of representative tumors from each individual group at day 16. (**E**) Tumors were resected and fixed in formalin according to standard operating procedure. Thereafter, they were embedded in paraffin, cut and stained with conventional H.E. staining. A scale bar accompanies each image (200 μm). Ctrl: Vehicle, ABT: ABT263. Crizo: Crizotinib, ABT + Crizo: ABT263 + Crizotinib. (**F**) The tumors were stained with TUNEL and photographs were taken. Scale bar indicates 50 μm.

After establishment of tumors, randomization was performed and four groups of animals with similar tumor sizes were assigned: (A) Vehicle, (B) ABT263, (C) Crizotinib, (D) ABT263 + Crizotinib. We found that host animals that received the combination treatment had significantly smaller tumors than animals receiving vehicle, ABT263 or Crizotinib (p < 0.05) (Fig. 6A,C,D). Amongst the treatment groups, we did not find a reduction in body weight after treatment (compared to the weight when treatment was initiated) (Fig. 6B). These findings are consistent with our *in vitro* observations and intimate that the combination treatment is reasonably tolerated and active *in vivo*. On the histopathological level, we found that the combination treatment reduced cellular density and the amount of mitosis, whereas the amount of necrosis was increased (Fig. 6E). In alignment with these findings, we found enhanced TUNEL staining in the combination treatment of ABT263 and Crizotinib (Fig. 6F). These observations are in keeping with the gross appearance of the tumors.

## Discussion

The heterogeneity of glioblastomas is an eminent challenge that calls for the simultaneous inhibition of several up-regulated pathways in these neoplasms^14,15^. The anti-apoptotic Bcl-2 family members represent an example of a class of proteins that is elevated in human glial tumors by several mechanisms. For instance, the Mcl-1 transcript has been shown to be increased and this is likely governed by gene amplification in a subset of cases. Discovered after Bcl-2 in the early 1990’s, Mcl-1 is a hallmark protein, driving resistance towards intrinsic apoptosis^16^. Binding to BAK, Mcl-1 inhibits mitochondrial outer membrane permeabilization and release of cytochrome-c into the cytosol. In the event that Bcl-xL and Bcl-2 are inhibited, Mcl-1 absorbs BAK to counteract cell death. While Mcl-1 is pivotal for survival of cancer cells, more recently its localization in the mitochondrial matrix was uncovered, modulating directly OXPHOS and helping to drive oxygen dependent ATP production^17^.

c-MET is a key regulator of glioma stem cells^2,3,18,19^. In this context, GBM sphere cultures displayed enhanced phosphorylation of c-MET, which mediates the transcription factor regulated reprogramming required for a “stem-cell” state. Not surprisingly, ectopic expression of c-MET inhibits differentiation of stem-like GBM cells in a Nanog dependent manner, confirming the pivotal role of this kinase pathway for tumor stem cells^3^. Our present findings in this report suggest that c-MET regulates the expression levels of Mcl-1 protein in model systems of human GBM. While GBM stem-like cells display high levels of c-MET, it has been known for some time that they have elevated levels of Mcl-1 as well. About a decade ago, researchers found that glioma stem-like cells are intrinsically resistant towards the first high-affinity BH3-mimetic, ABT737. This resistance was convincingly linked to higher Mcl-1 levels in stem-like GBM cells as compared to the differentiated counterparts^20^. Therefore, our findings establish an important link between c-MET signaling and Mcl-1 in this critical cell population and suggest that interference with c-MET is a novel means to suppress Mcl-1 levels.

To understand the link between c-MET signaling and Mcl-1 regulation, we focused on tumor cell metabolism. Our hypothesis was that c-MET inhibition in the presence or absence of Bcl-2/Bcl-xL inhibition leads to suppression of oxygen consumption rate and ATP production, which in turn negatively affects protein synthesis and levels of short-lived proteins, such as Mcl-1. Consistently, we provide evidence that c-MET inhibition slows ATP production and ATP levels and decreases protein synthesis. To the best of our knowledge, these observations are novel in the context of GBM and should be seen in connection with other kinase inhibitors, such as PI3K that akin to c-MET inhibitors suppress energy metabolism of tumor cells. The fact that these kinase inhibitors interfere with both glycolysis and OXPHOS is appealing because inhibition of one selective energetic pathway very commonly leads to compensatory activation of the other. In essence, these metabolic vulnerabilities are exploitable for tumor combination therapies. Other examples of this kind are the dependency on glutamine metabolism of c-myc driven cell cultures or IDH1 mutated cells^21–23^. In this context, our own group has recently made the discovery that mutated IDH1 renders cells susceptible to Bcl-xL inhibition, which suggests that patients with the IDH1 R132H mutation might benefit from the addition of ABT263 to their treatment regimen^12^. Although we have not specifically addressed this issue in this study it is possible that patients with high c-MET levels might not only benefit from the addition of Crizotinib like compounds to their regimen, but also from the implementation of BH3-mimetics.

The significance of suppressing Mcl-1 levels^6,10,24–32^ is obvious based on our elaborations above. In keeping with the expectation, c-MET inhibition along with dual Bcl-2/Bcl-xL inhibition causes synthetic lethality in stem-like and established GBM cells, which is pharmacologically exemplified by the drug combination of ABT263 and Crizotinib, two drugs that are tested or used in patients already. To validate this approach *in vivo*, we utilized a PDX model system of human GBM and confirmed our hypothesis that the combination treatment is more efficacious than the single treatments. These results are a foundation to consider this combination treatment for further patient related development.

## Materials and Methods

### Reagents

ABT263, WEHI-539, A1210477, ABT199 and Crizotinib were purchased from Sellekchem (New York, NY).

### Cell cultures and growth conditions

U87MG and LN229 human glioblastoma cell lines were obtained from the American Type Culture Collection (Manassas, VA). NCH644 stem cell-like glioma cells were purchased from Cell Line Services (CLS, Heidelberg, Germany). The respective cell line depository authenticated the cells. All cell lines were obtained between 2013–2015 and cultured as previously described in^12,33–41^.

### Cell viability assays

Viability assays were performed as previously described in^12,33–41^.

### Measurement of apoptosis and mitochondrial membrane potential

For Annexin V/propidium iodide staining, the Annexin V Apoptosis Detection Kit (BD Pharmingen) was used as previously described^12,33–41^. Tetramethylrhodamine, ethyl-ester (TMRE) staining was performed according to the manufacturer’s instructions (Mitochondrial Membrane Potential kit, Cell Signaling Technology, Danvers, MA). The data were analyzed with the FlowJo software (version 8.7.1; Tree Star, Ashland, OR).

### Extracellular flux analysis

Extracellular flux analysis was performed on the Seahorse XFe24 device (Agilent, Inc.). Approximately 40,000 LN229 GBM cells were seeded on each well of the XFe24 plate. The mitochondrial stress assay was performed as described in^12^.

### Transfections of siRNAs

Transfections with the respected oligonucleotides were performed as previously described in^12,33–41^.

### Western blot analysis and protein capillary electrophoresis

Specific protein expression in cell lines was determined by Western blot analysis or capillary electrophoresis as described before^27,33^. Capillary electrophoresis on the fully automated Wes instrument (Proteinsimple, San Jose, CA) represents a highly-innovative and sensitive approach (high-dynamic range) to measure protein levels in tissue or cells. All the antibodies were purchased from Cell Signaling Technology.

### Subcutaneous xenograft model

GBM12 PDX cells were implanted subcutaneously into the flanks of 6–8 week-old SCID SHO mice as described before^12,33–41^. Measurements were performed with a caliper and tumor sizes were calculated as (length × width^2^)/2. Treatment was performed intraperitoneally three times a week. For intraperitoneal application Crizotinib and ABT263 were dissolved in 10% DMSO, 32% Cremophor EL (SIGMA, St. Louis, MO), 8% Ethanol (Pharmco-Aaper, Brookfield,CT) and 50% PBS. At the end of the experiment gross images were taken as earlier described^12,33–41^. Histology was performed as described earlier^12,33–41^.

### Real-time PCR and cDNA synthesis

cDNA synthesis and RT-PCR were performed as described before. Primer sequences for 18 S, Mcl-1 and PMAIP1 (Noxa) are described elsewhere^12,33–41^.

### Statistical analysis

Statistical significance was assessed by Student’s t-test using Prism version 7.00 (GraphPad, La Jolla, CA). 3 replicates were performed unless otherwise described. A p ≤ 0.05 was considered statistically significant. The CompuSyn software (ComboSyn, Inc., Paramus, NJ, U.S.A.) was used to calculate drug synergy^12,33–42^.

### Ethical approval

All procedures were in accordance with Animal Welfare Regulations and approved by the Institutional Animal Care and Use Committee at the Columbia University Medical Center.

### Data availability statement

All data generated or analyzed during this study are included in this published article (and its Supplementary Information files).

## Electronic supplementary material

Supplementary Dataset 1

Uncropped Gel Images
