# Supplementary material for: Inhibition of Bcl-2/Bcl-xL and c-MET causes synthetic lethality in model systems of glioblastoma
Source: Sci Rep. 2018 May 9;8:7373. doi: 10.1038/s41598-018-25802-0 (PMC5943348; doi:10.1038/s41598-018-25802-0)
Supplement: Supplementary file 1 — Supplementary Dataset 1 [file 41598_2018_25802_MOESM1_ESM.docx]

Inhibition of Bcl-2/Bcl-xL and c-MET causes synthetic lethality in model systems of glioblastoma

Yiru Zhang^1^, Chiaki Tsuge Ishida^1^, Chang Shu^1^, Giulio Kleiner^2^, Maria J. Sanchez-Quintero ^2^, Elena Bianchetti^1^, Catarina M. Quinzii^2^, Mike-Andrew Westhoff ^4^, Georg Karpel-Massler^3^ and Markus D. Siegelin^1^

**Supplementary Figures and Legends:**

**
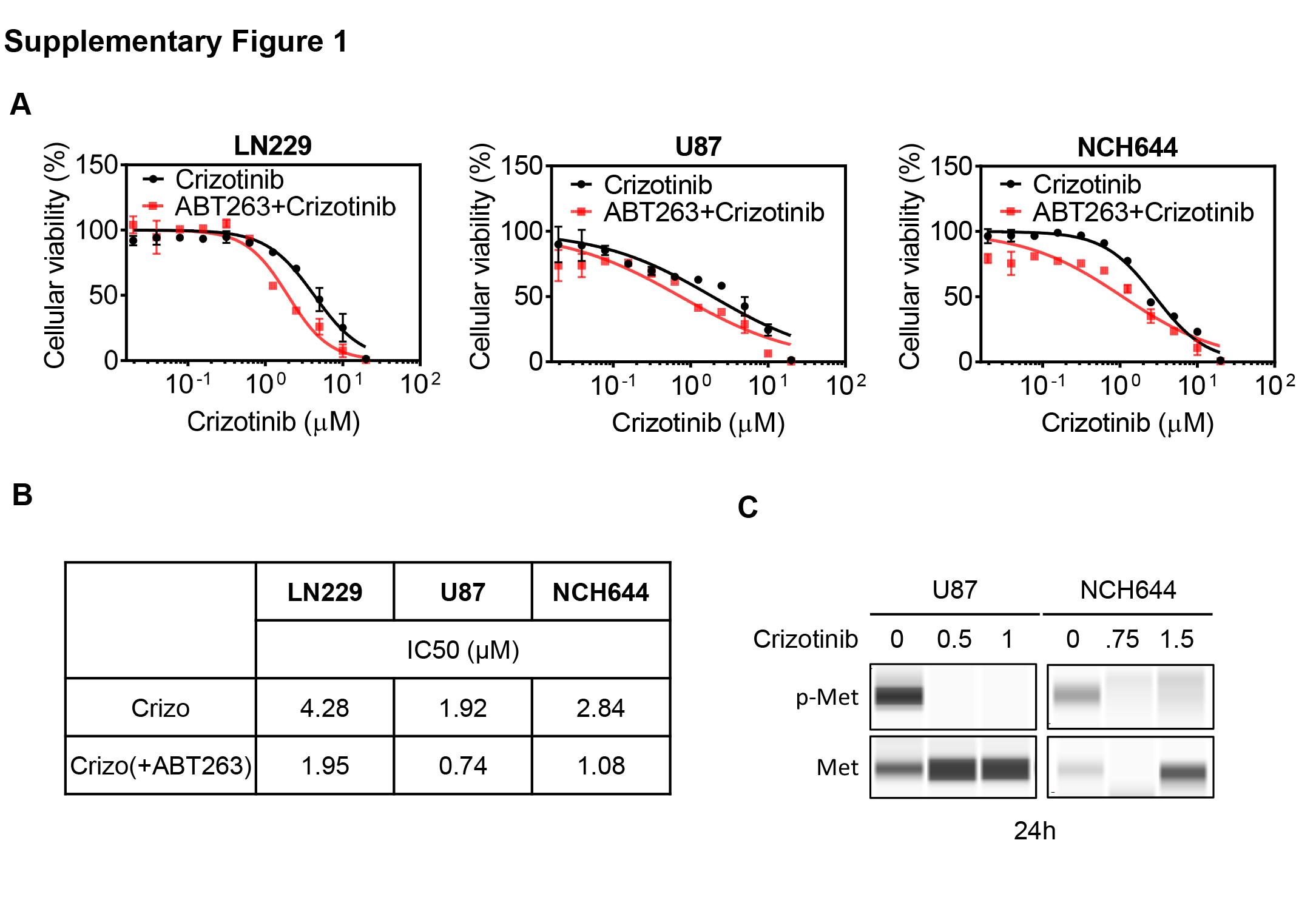
**

**Supplementary Figure 1: The combination treatment of ABT263 and Crizotinib elicits a synergistic reduction in growth of glioblastoma cultures**

**A, B**, LN229, U87 and NCH644 GBM stem-like cells were treated with Crizotinib in the presence or absence of ABT263 for 72h and analyzed by CellTiter Glo assay. Using non-linear regression analysis, IC_50_ values were determined and are displayed in B. **C**, U87 and NCH644 GBM cells were treated with increasing concentrations of Crizotinib. Whole cell protein lysates were collected and analyzed by capillary electrophoresis, using antibodies against phosphorylated MET and total MET (on-target confirmation). Concentrations are in μM.

**
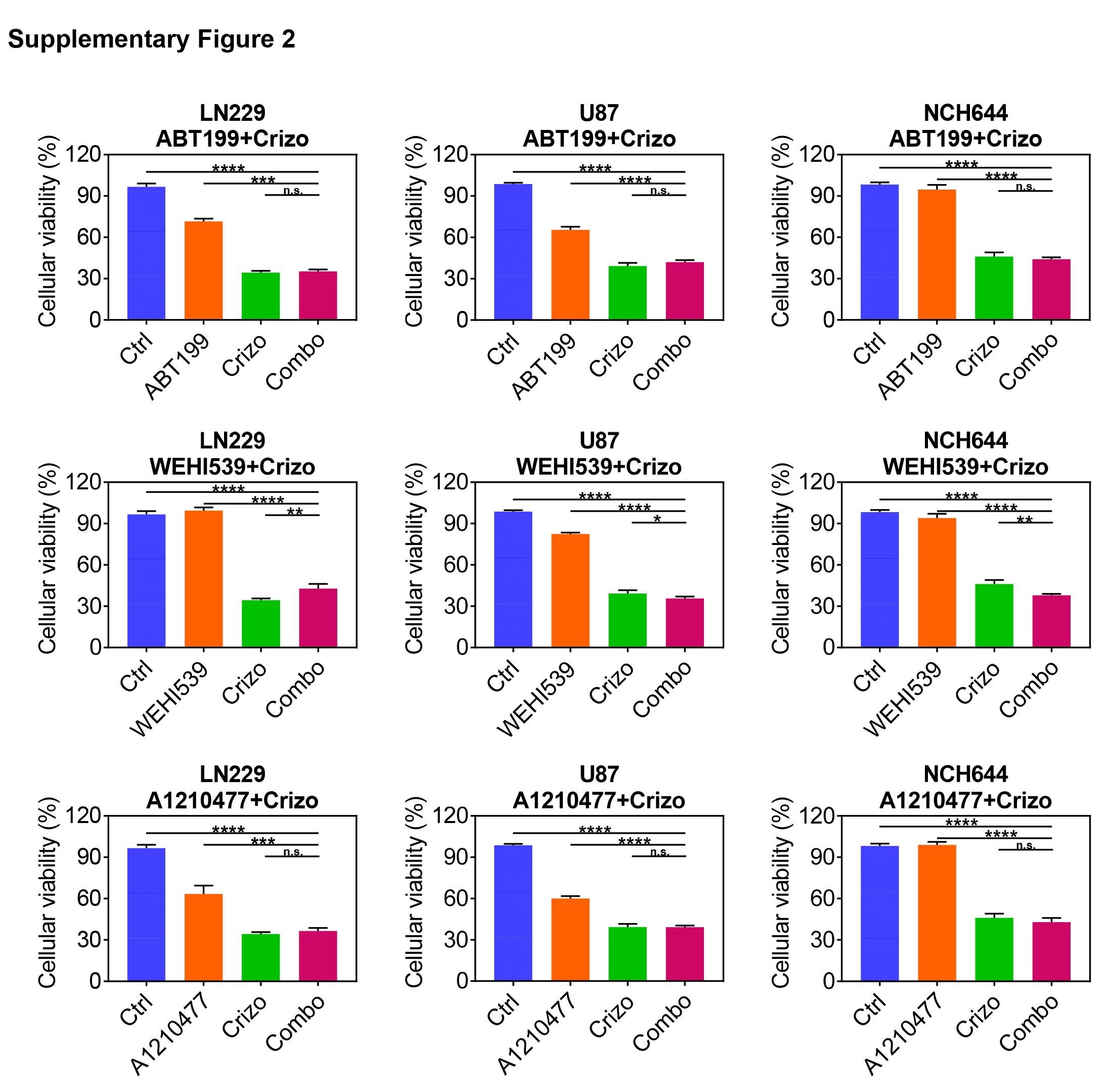
**

**Supplementary Figure 2: Inhibition of both Bcl-2 and Bcl-xL is required for the synergistic interaction of ABT263 and Crizotinib**

LN229, U87 or NCH644 cells were treated with selective BH3-mimetics, ABT199 (Bcl-2 inhibitor), WEHI539 (Bcl-xL inhibitor), or A1210477 (Mcl-1 inhibitor) in the presence or absence of Crizotinib for 72h. Thereafter, cell viability was analyzed and compared.

**
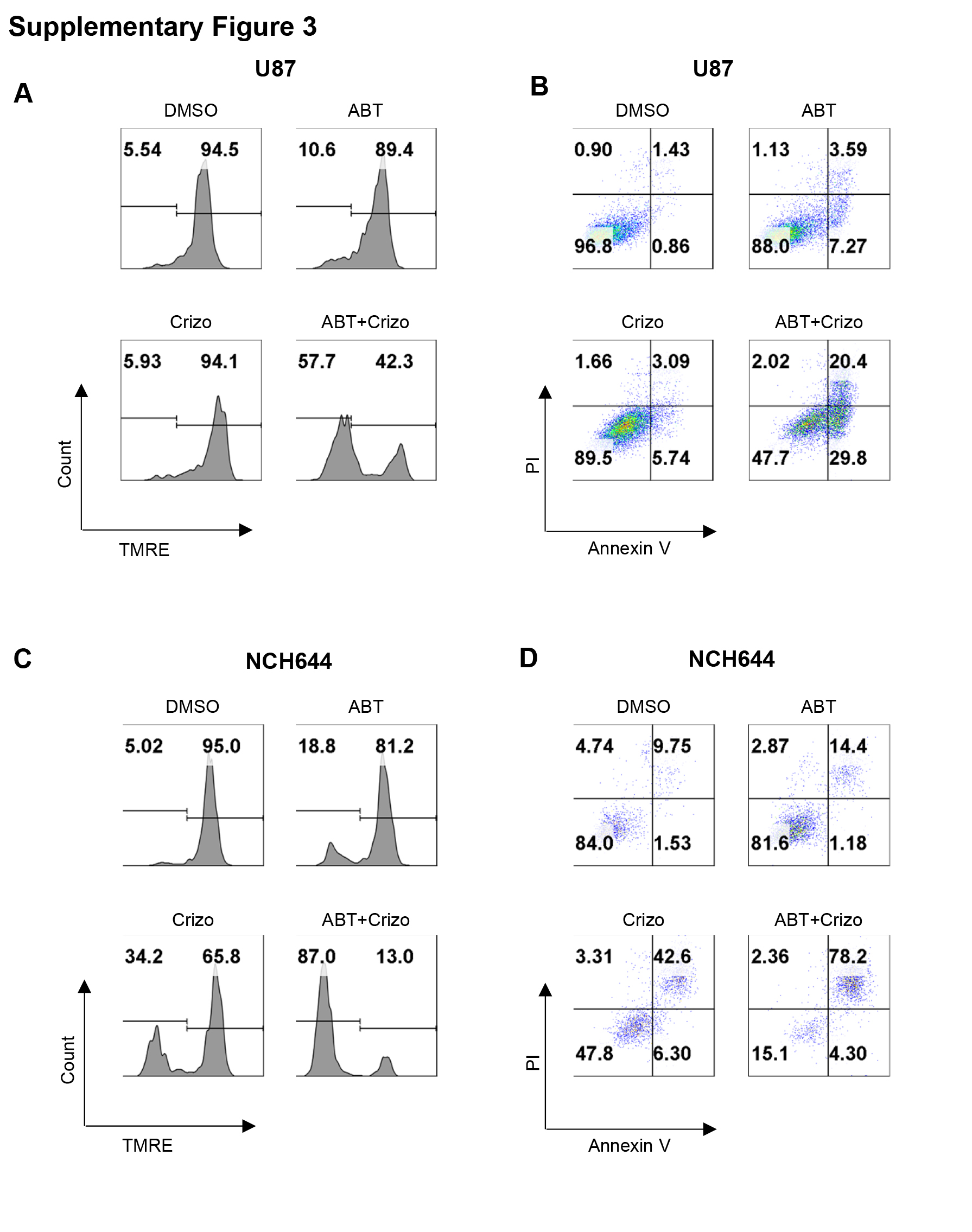
**

**Supplementary Figure 3: The combination treatment of ABT263 and Crizotinib leads to enhanced induction of apoptosis and loss of mitochondrial membrane potential**

**A**, U87 GBM cells were treated with ABT263 (ABT), Crizotinib (Crizo) or the combination of both and stained with TMRE and analyzed by flow cytometry. **B**, Alternatively, cells were labeled with Annexin V/propidium iodide and subjected to multi-parametric flow cytometry. **C**, NCH644 GBM stem-like cells were treated with ABT263 (ABT), Crizotinib (Crizo) or the combination of both and stained with TMRE and analyzed by flow cytometry. **D**, Alternatively, cells were labeled with Annexin V/propidium iodide and subjected to multi-parametric flow cytometry.


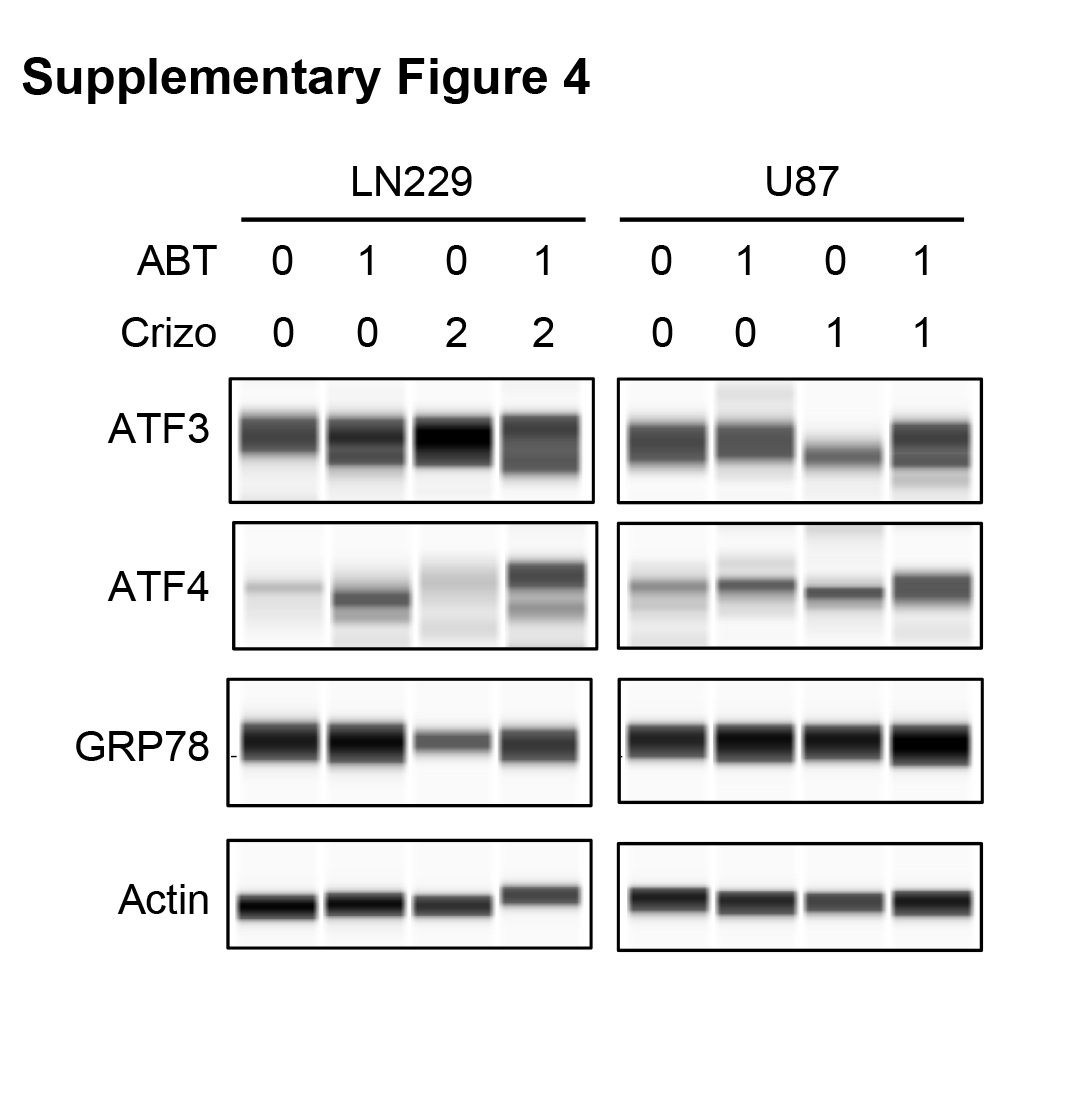


**Supplementary Figure 4: The combination treatment of ABT263 and Crizotinib elicits an induction of an endoplasmic reticulum stress response (ER).**

LN229 and U87 GBM cells were treated with ABT263 (ABT) or Crizotinib (Criz) as indicated for 24h. Concentrations are in μM. Thereafter, whole cell protein lysates were collected and analyzed by capillary electrophoresis for the expression of ATF3, ATF4, GRP78 and Actin.
