# Supplementary material for: Inhibition of Bcl-2/Bcl-xL and c-MET causes synthetic lethality in model systems of glioblastoma
Source: Sci Rep. 2018 May 9;8:7373. doi: 10.1038/s41598-018-25802-0 (PMC5943348; doi:10.1038/s41598-018-25802-0)

Inhibition of Bcl-2/Bcl-xL and c-MET causes synthetic lethality in model systems of glioblastoma

Yiru Zhang[1], Chiaki Tsuge Ishida[1], Chang Shu[1], Giulio Kleiner[2], Maria J. Sanchez-Quintero [2], Elena Bianchetti[1], Catarina M. Quinzii[2], Mike-Andrew Westhoff [4], Georg Karpel-Massler[3] and Markus D. Siegelin[1]

Uncropped gel and capillary electrophoresis images

Figure 2F

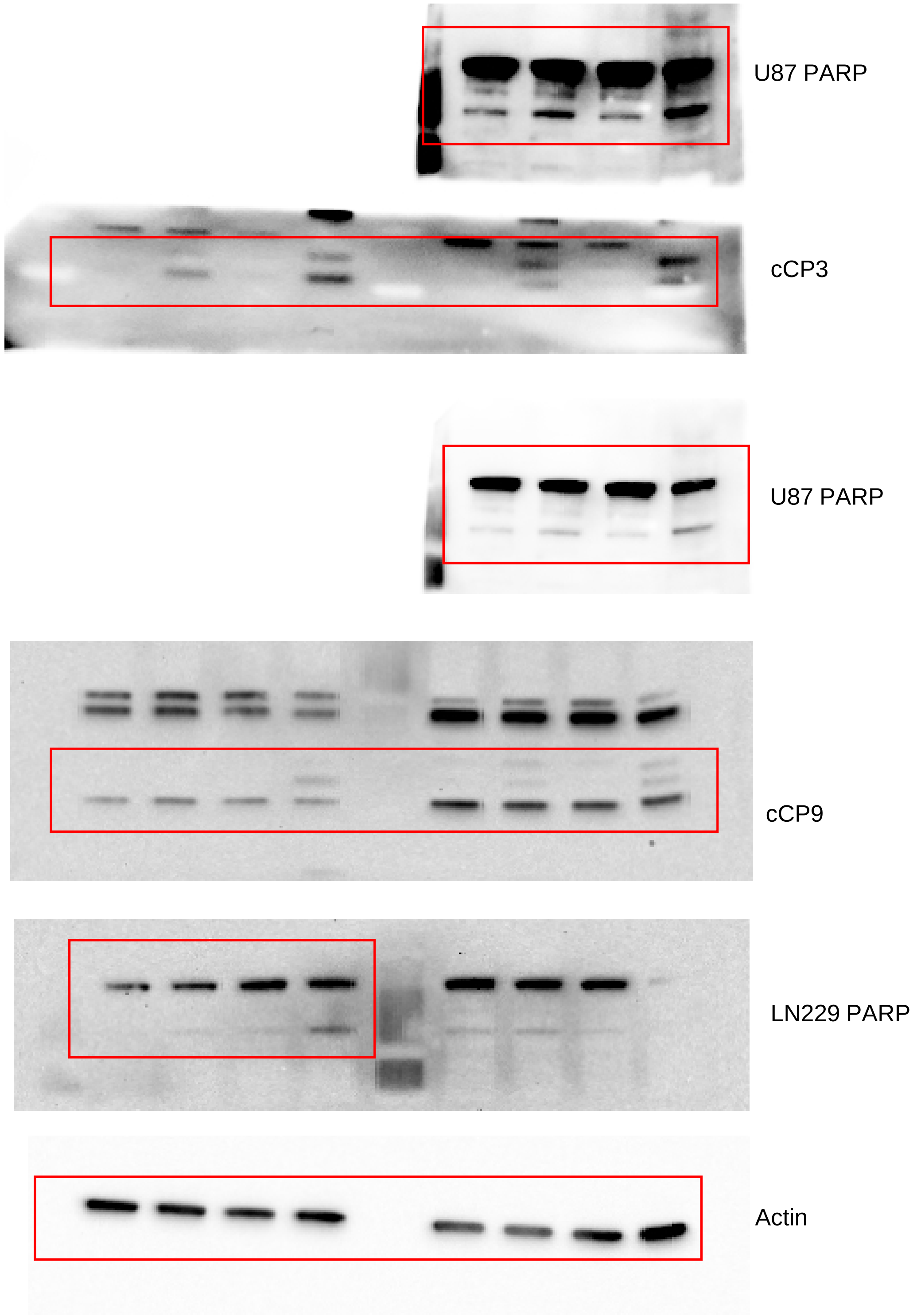

Figure 3A

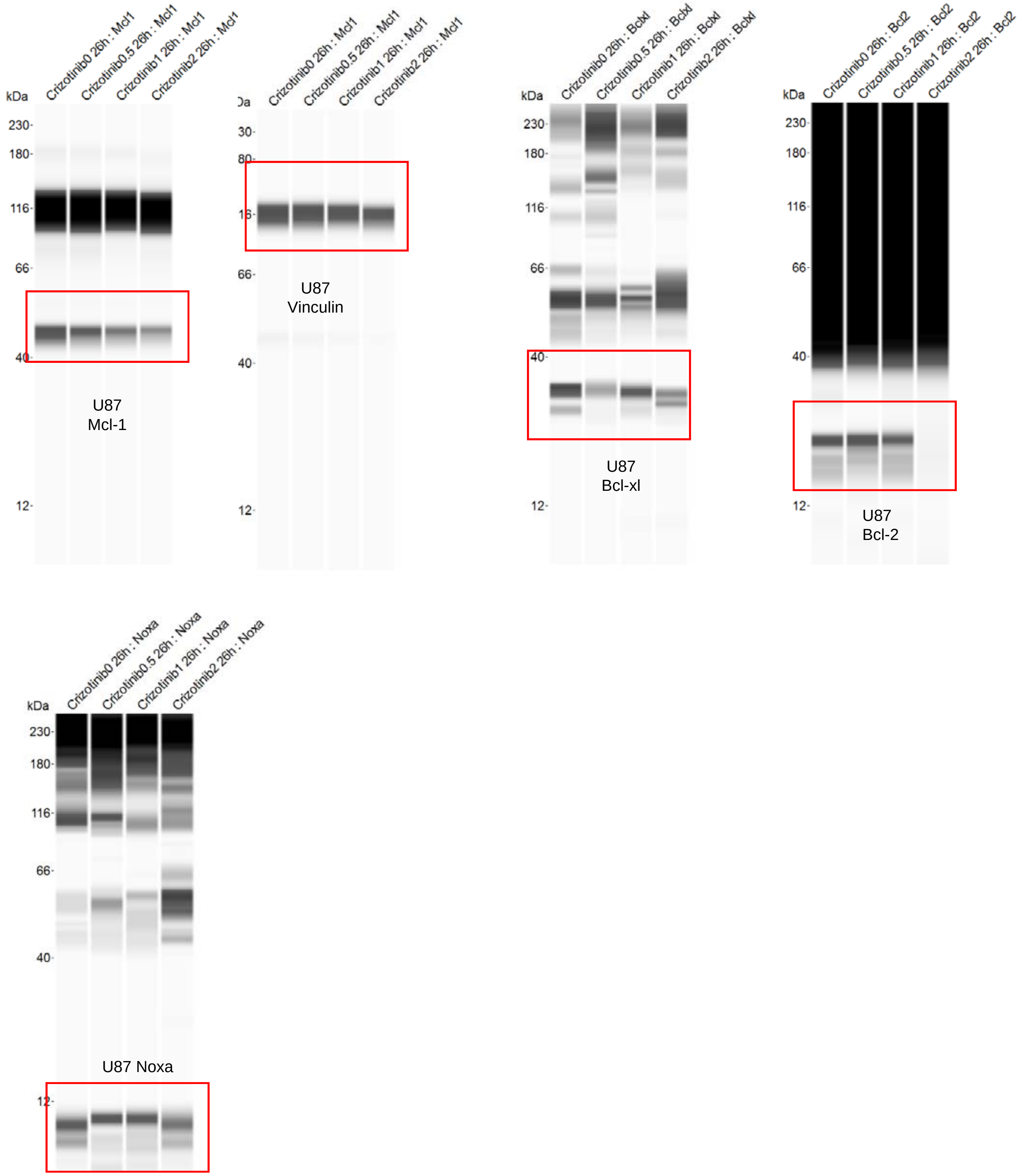
kDa
Crizotinib0 26h : Mcl1
Crizotinib0.5 26h : Mcl1
Crizotinib1 26h : Mcl1
Crizotinib2 26h : Mcl1
230
180
116
66
40
12
U87
Mcl-1
Crizotinib0 26h : Mcl1
Crizotinib0.5 26h : Mcl1
Crizotinib1 26h : Mcl1
Crizotinib2 26h : Mcl1
66
40
12
U87
Vinculin
kDa
Crizotinib0 26h : Bclxl
Crizotinib0.5 26h : Bclxl
Crizotinib1 26h : Bclxl
Crizotinib2 26h : Bclxl
230
180
116
66
40
12
U87
Bcl-xl
kDa
Crizotinib0 26h : Bcl2
Crizotinib0.5 26h : Bcl2
Crizotinib1 26h : Bcl2
Crizotinib2 26h : Bcl2
230
180
116
66
40
12
U87
Bcl-2
kDa
Crizotinib0 26h : Noxa
Crizotinib0.5 26h : Noxa
Crizotinib1 26h : Noxa
Crizotinib2 26h : Noxa
230
180
116
66
40
12
U87 Noxa

Figure 3A

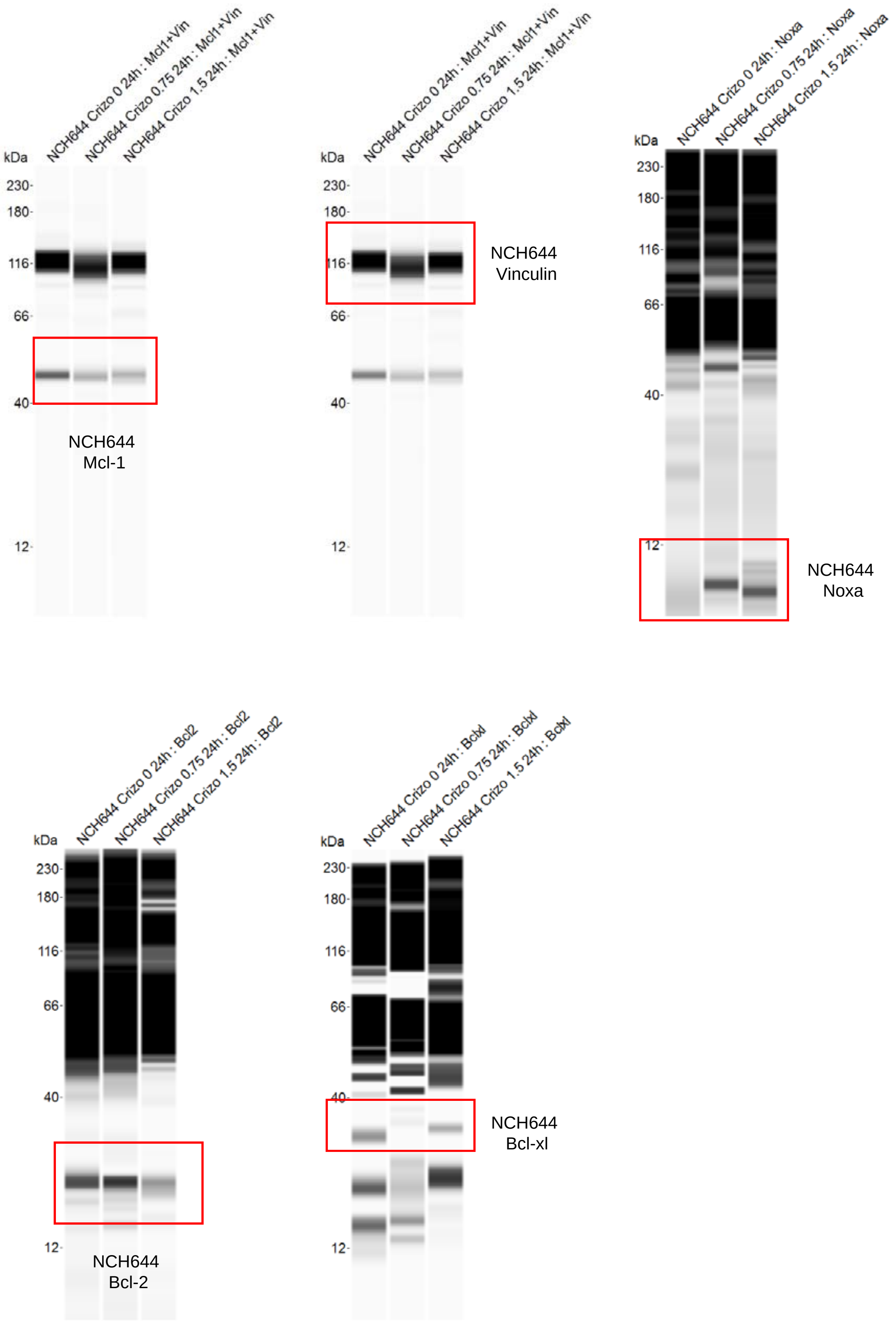

NCH644 Crizo 0 24h : Mcl1+Vin
NCH644 Crizo 0.75 24h : Mcl1+Vin
NCH644 Crizo 1.5 24h : Mcl1+Vin
kDa
230
180
116
66
40
12
NCH644
Mcl-1
NCH644
Vinculin
NCH644 Crizo 0 24h : Noxa
NCH644 Crizo 0.75 24h : Noxa
NCH644 Crizo 1.5 24h : Noxa
NCH644
Noxa
NCH644 Crizo 0 24h : Bcl2
NCH644 Crizo 0.75 24h : Bcl2
NCH644 Crizo 1.5 24h : Bcl2
NCH644
Bcl-2
NCH644 Crizo 0 24h : Bclxl
NCH644 Crizo 0.75 24h : Bclxl
NCH644 Crizo 1.5 24h : Bclxl
NCH644
Bcl-xl

Figure 3A

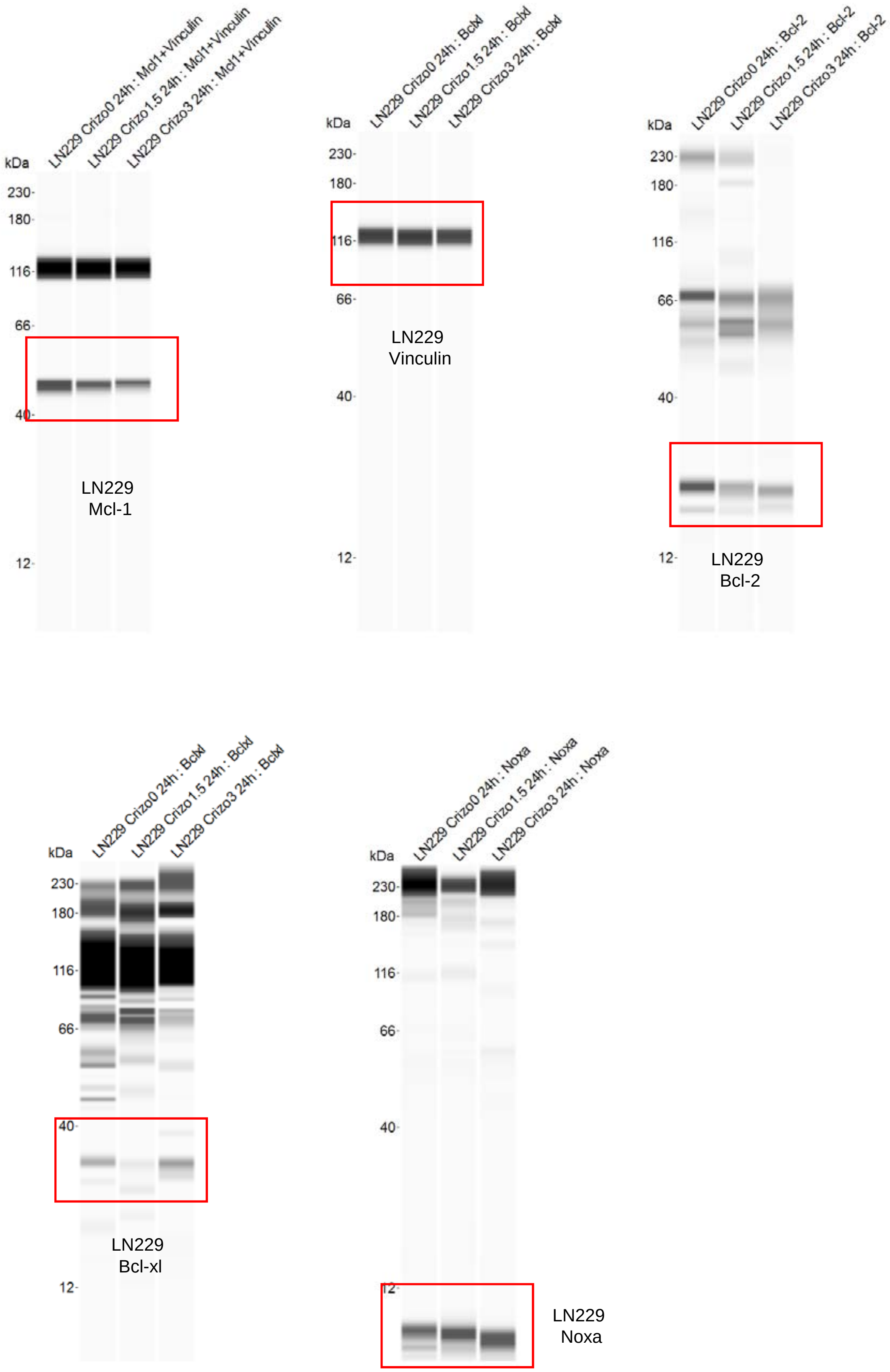

LN229 Crizo0 24h : Mcl1+Vinculin
LN229 Crizo1.5 24h : Mcl1+Vinculin
LN229 Crizo3 24h : Mcl1+Vinculin
kDa
230
180
116
66
40
12
LN229
Mcl-1
LN229 Crizo0 24h : Bclxl
LN229 Crizo1.5 24h : Bclxl
LN229 Crizo3 24h : Bclxl
kDa
230
180
116
66
40
12
LN229
Vinculin
LN229 Crizo0 24h : Bcl-2
LN229 Crizo1.5 24h : Bcl-2
LN229 Crizo3 24h : Bcl-2
kDa
230
180
116
66
40
12
LN229
Bcl-2
LN229 Crizo0 24h : Bclxl
LN229 Crizo1.5 24h : Bclxl
LN229 Crizo3 24h : Bclxl
kDa
230
180
116
66
40
12
LN229
Bcl-xl
LN229 Crizo0 24h : Noxa
LN229 Crizo1.5 24h : Noxa
LN229 Crizo3 24h : Noxa
kDa
230
180
116
66
40
12
LN229
Noxa

Figure 3B

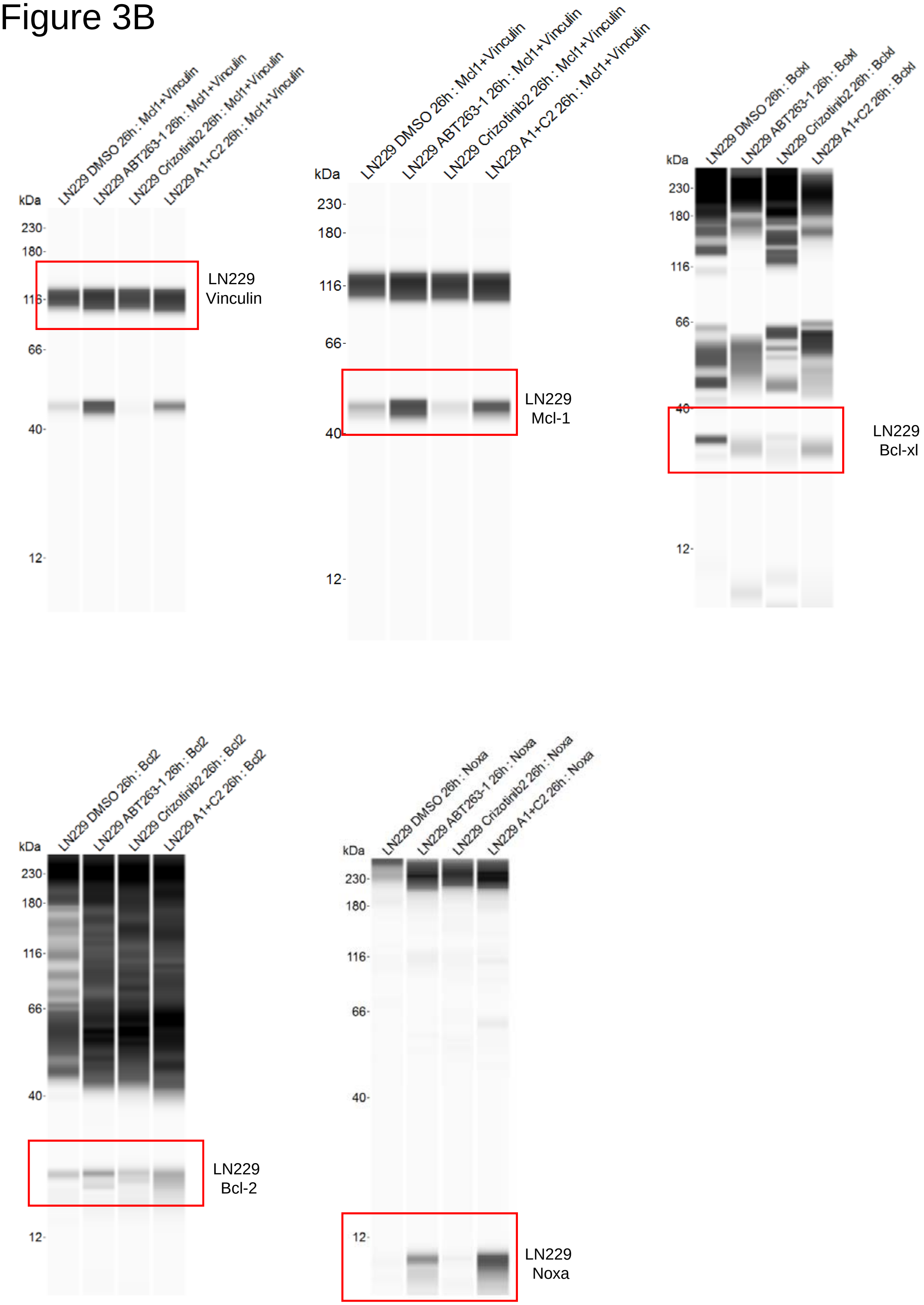

LN229 DMSO 26h : Mcl1+Vinculin
LN229 ABT263-1 26h : Mcl1+Vinculin
LN229 Crizotinib2 26h : Mcl1+Vinculin
LN229 A1+C2 26h : Mcl1+Vinculin
kDa
230
180
116
66
40
12
LN229
Vinculin
LN229
Mcl-1
LN229 DMSO 26h : Bclxl
LN229 ABT263-1 26h : Bclxl
LN229 Crizotinib2 26h : Bclxl
LN229 A1+C2 26h : Bclxl
LN229
Bcl-xl
LN229 DMSO 26h : Bcl2
LN229 ABT263-1 26h : Bcl2
LN229 Crizotinib2 26h : Bcl2
LN229 A1+C2 26h : Bcl2
LN229
Bcl-2
LN229 DMSO 26h : Noxa
LN229 ABT263-1 26h : Noxa
LN229 Crizotinib2 26h : Noxa
LN229 A1+C2 26h : Noxa
LN229
Noxa

Figure 3B

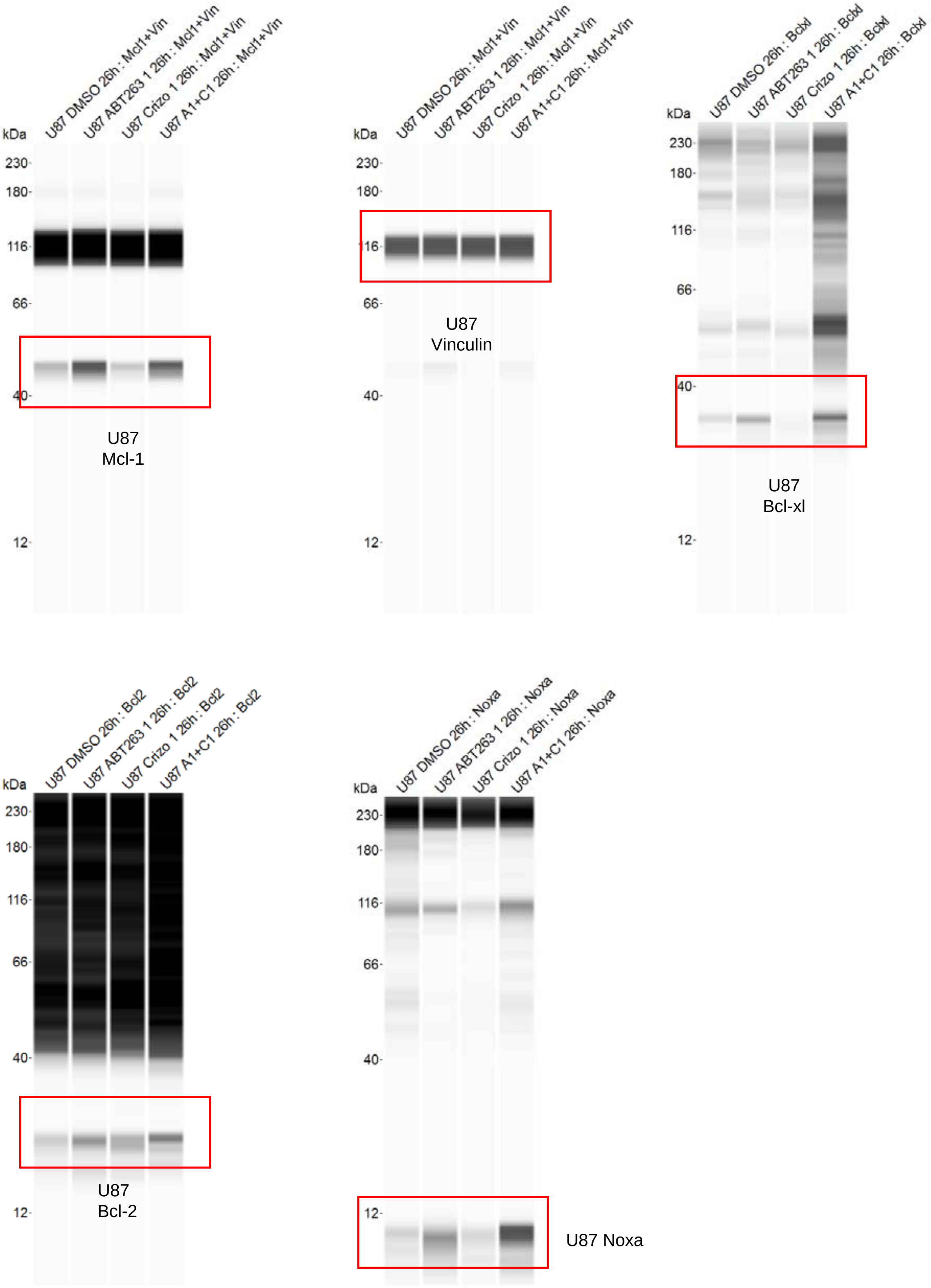
U87 DMSO 26h : Mcl1+Vin
U87 ABT263 1 26h : Mcl1+Vin
U87 Crizo 1 26h : Mcl1+Vin
U87 A1+C1 26h : Mcl1+Vin
kDa
230
180
116
66
40
12
U87
Mcl-1
U87
Vinculin
U87 DMSO 26h : Bclxl
U87 ABT263 1 26h : Bclxl
U87 Crizo 1 26h : Bclxl
U87 A1+C1 26h : Bclxl
U87
Bcl-xl
U87 DMSO 26h : Bcl2
U87 ABT263 1 26h : Bcl2
U87 Crizo 1 26h : Bcl2
U87 A1+C1 26h : Bcl2
U87
Bcl-2
U87 DMSO 26h : Noxa
U87 ABT263 1 26h : Noxa
U87 Crizo 1 26h : Noxa
U87 A1+C1 26h : Noxa
U87 Noxa

Figure 3E

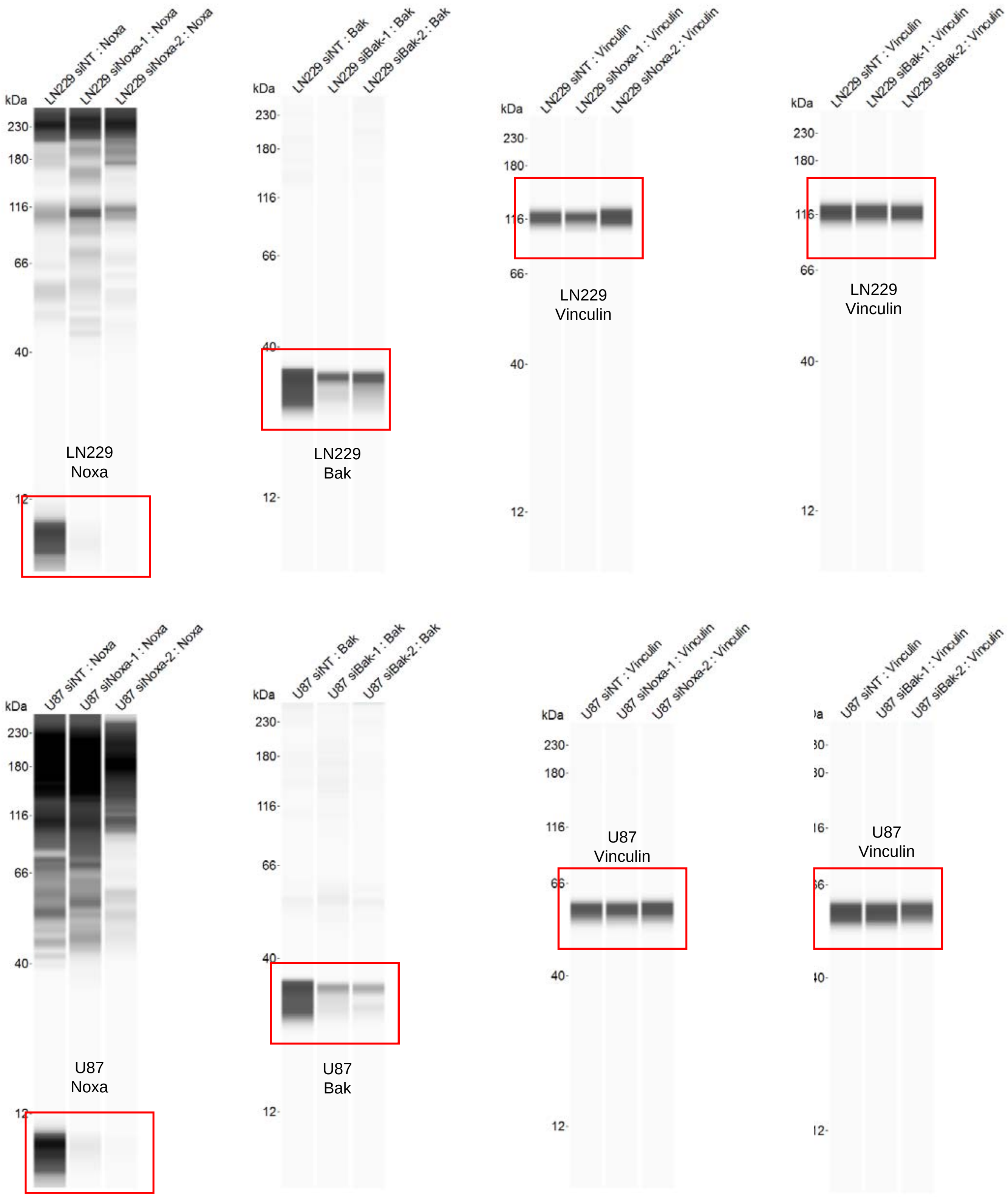
LN229 siNT : Noxa
LN229 siNoxa-1 : Noxa
LN229 siNoxa-2 : Noxa
kDa
230
180
116
66
40
12
LN229
Noxa
LN229 siNT : Bak
LN229 siBak-1 : Bak
LN229 siBak-2 : Bak
LN229
Bak
LN229 siNT : Vinculin
LN229 siNoxa-1 : Vinculin
LN229 siNoxa-2 : Vinculin
LN229
Vinculin
LN229 siNT : Vinculin
LN229 siBak-1 : Vinculin
LN229 siBak-2 : Vinculin
LN229
Vinculin
U87 siNT : Noxa
U87 siNoxa-1 : Noxa
U87 siNoxa-2 : Noxa
U87
Noxa
U87 siNT : Bak
U87 siBak-1 : Bak
U87 siBak-2 : Bak
U87
Bak
U87 siNT : Vinculin
U87 siNoxa-1 : Vinculin
U87 siNoxa-2 : Vinculin
U87
Vinculin
U87 siNT : Vinculin
U87 siBak-1 : Vinculin
U87 siBak-2 : Vinculin
U87
Vinculin

Figure 3E

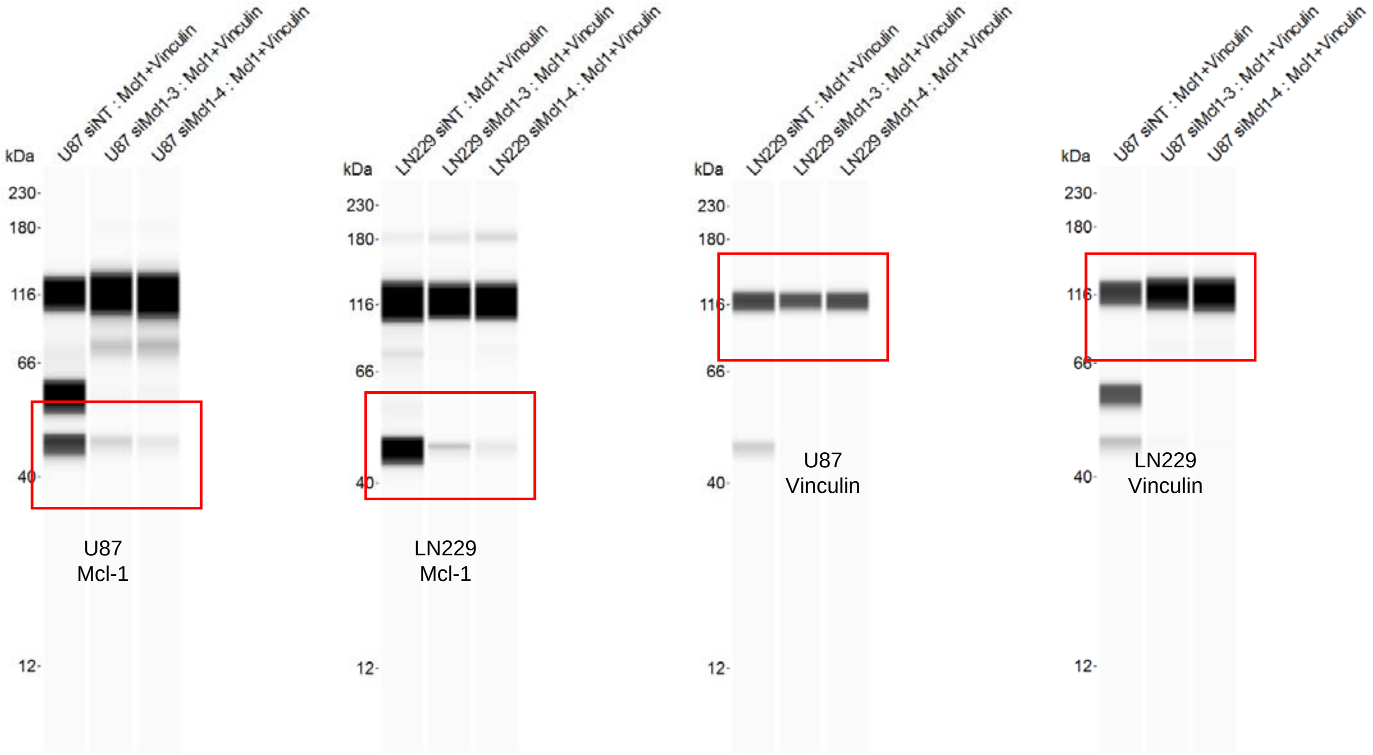

Figure 4D

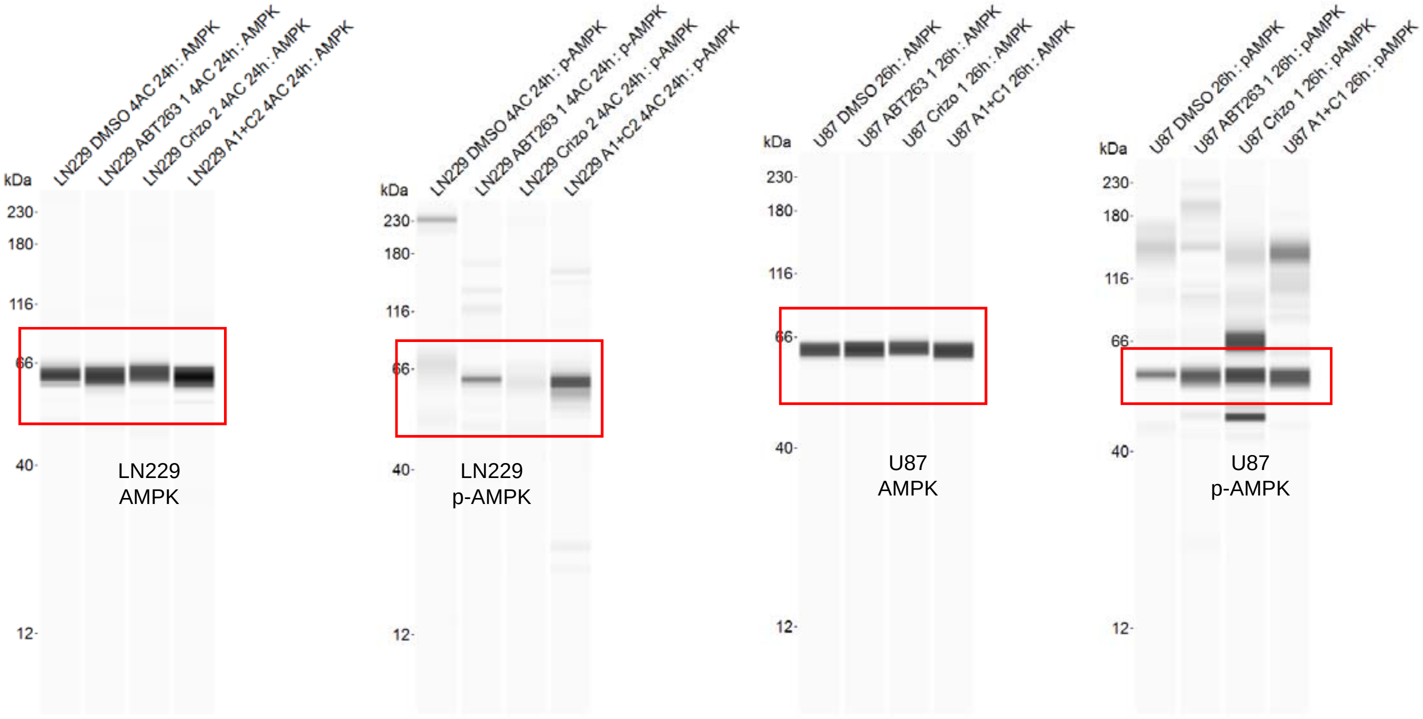

Figure 5A

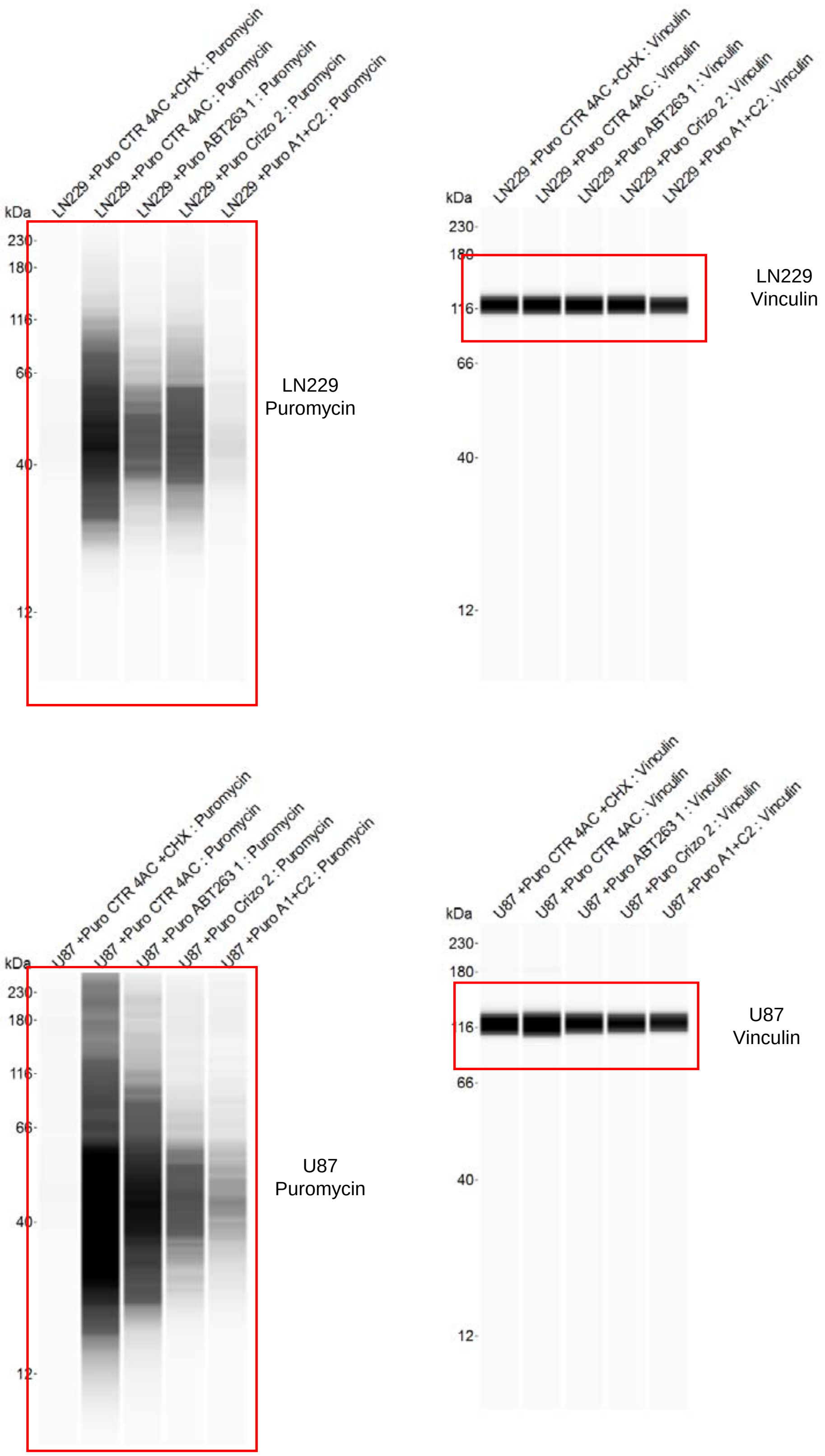

LN229 +Puro CTR 4AC +CHX : Puromycin
LN229 +Puro CTR 4AC : Puromycin
LN229 +Puro ABT263 1 : Puromycin
LN229 +Puro Crizo 2 : Puromycin
LN229 +Puro A1+C2 : Puromycin
kDa
230
180
116
66
40
12
LN229
Puromycin
LN229 +Puro CTR 4AC +CHX : Vinculin
LN229 +Puro CTR 4AC : Vinculin
LN229 +Puro ABT263 1 : Vinculin
LN229 +Puro Crizo 2 : Vinculin
LN229 +Puro A1+C2 : Vinculin
kDa
230
180
116
66
40
12
LN229
Vinculin
U87 +Puro CTR 4AC +CHX : Puromycin
U87 +Puro CTR 4AC : Puromycin
U87 +Puro ABT263 1 : Puromycin
U87 +Puro Crizo 2 : Puromycin
U87 +Puro A1+C2 : Puromycin
kDa
230
180
116
66
40
12
U87
Puromycin
U87 +Puro CTR 4AC +CHX : Vinculin
U87 +Puro CTR 4AC : Vinculin
U87 +Puro ABT263 1 : Vinculin
U87 +Puro Crizo 2 : Vinculin
U87 +Puro A1+C2 : Vinculin
kDa
230
180
116
66
40
12
U87
Vinculin

Figure 5B

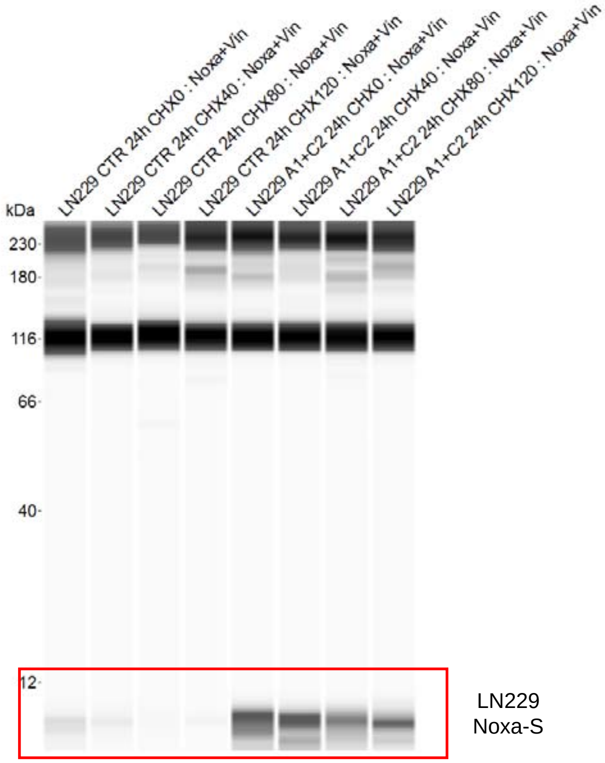

LN229 CTR 24h CHX0 : Noxa+Vin
LN229 CTR 24h CHX40 : Noxa+Vin
LN229 CTR 24h CHX80 : Noxa+Vin
LN229 CTR 24h CHX120 : Noxa+Vin
LN229 A1+C2 24h CHX0 : Noxa+Vin
LN229 A1+C2 24h CHX40 : Noxa+Vin
LN229 A1+C2 24h CHX80 : Noxa+Vin
LN229 A1+C2 24h CHX120 : Noxa+Vin
kDa
230
180
116
66
40
12
LN229
Noxa-S

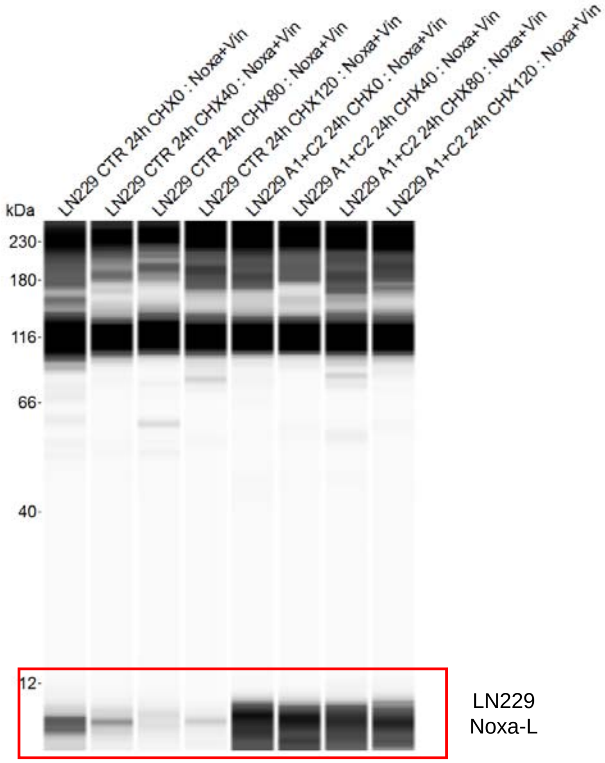

LN229 CTR 24h CHX0 : Noxa+Vin
LN229 CTR 24h CHX40 : Noxa+Vin
LN229 CTR 24h CHX80 : Noxa+Vin
LN229 CTR 24h CHX120 : Noxa+Vin
LN229 A1+C2 24h CHX0 : Noxa+Vin
LN229 A1+C2 24h CHX40 : Noxa+Vin
LN229 A1+C2 24h CHX80 : Noxa+Vin
LN229 A1+C2 24h CHX120 : Noxa+Vin
kDa
230
180
116
66
40
12
LN229
Noxa-L

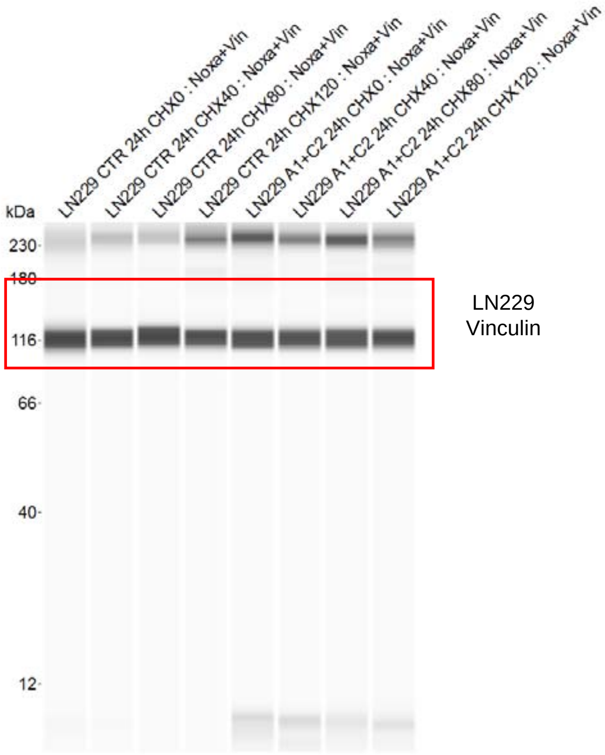

LN229 CTR 24h CHX0 : Noxa+Vin
LN229 CTR 24h CHX40 : Noxa+Vin
LN229 CTR 24h CHX80 : Noxa+Vin
LN229 CTR 24h CHX120 : Noxa+Vin
LN229 A1+C2 24h CHX0 : Noxa+Vin
LN229 A1+C2 24h CHX40 : Noxa+Vin
LN229 A1+C2 24h CHX80 : Noxa+Vin
LN229 A1+C2 24h CHX120 : Noxa+Vin
kDa
230
180
116
66
40
12
LN229
Vinculin

Figure 5B

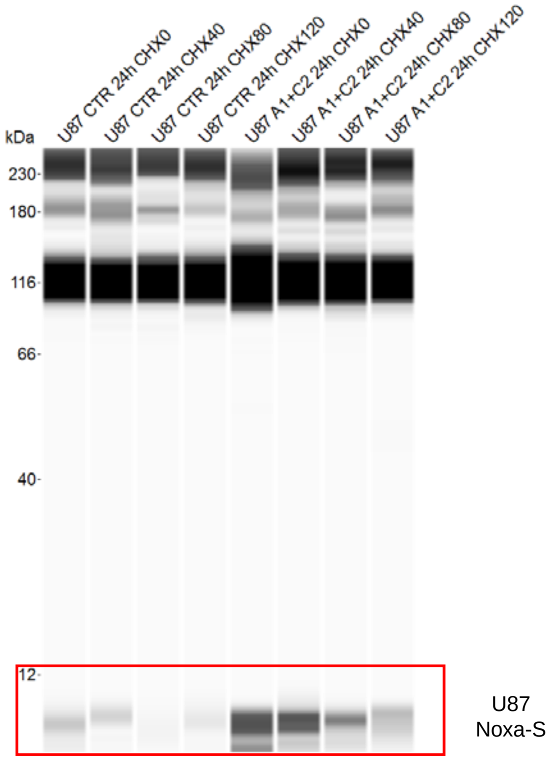
U87 CTR 24h CHX0
U87 CTR 24h CHX40
U87 CTR 24h CHX80
U87 CTR 24h CHX120
U87 A1+C2 24h CHX0
U87 A1+C2 24h CHX40
U87 A1+C2 24h CHX80
U87 A1+C2 24h CHX120
kDa
230
180
116
66
40
12
U87
Noxa-S

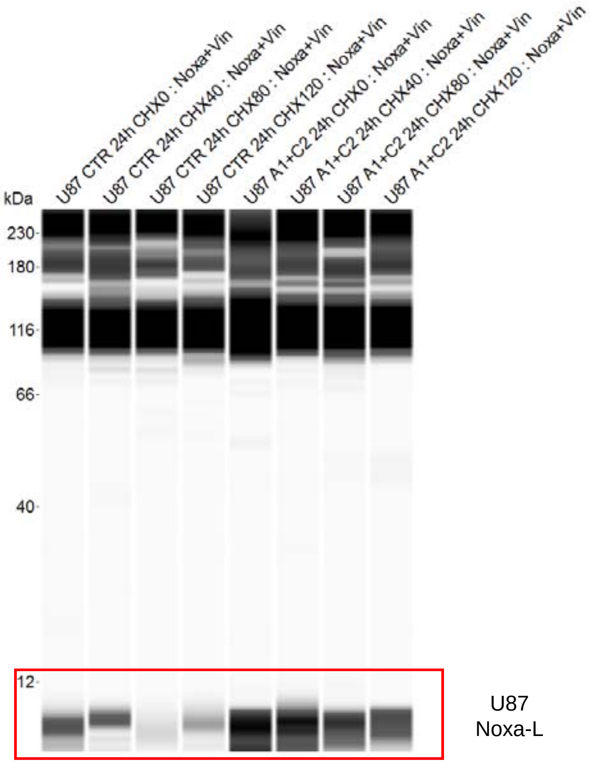
U87 CTR 24h CHX0 : Noxa+Vin
U87 CTR 24h CHX40 : Noxa+Vin
U87 CTR 24h CHX80 : Noxa+Vin
U87 CTR 24h CHX120 : Noxa+Vin
U87 A1+C2 24h CHX0 : Noxa+Vin
U87 A1+C2 24h CHX40 : Noxa+Vin
U87 A1+C2 24h CHX80 : Noxa+Vin
U87 A1+C2 24h CHX120 : Noxa+Vin
kDa
230
180
116
66
40
12
U87
Noxa-L

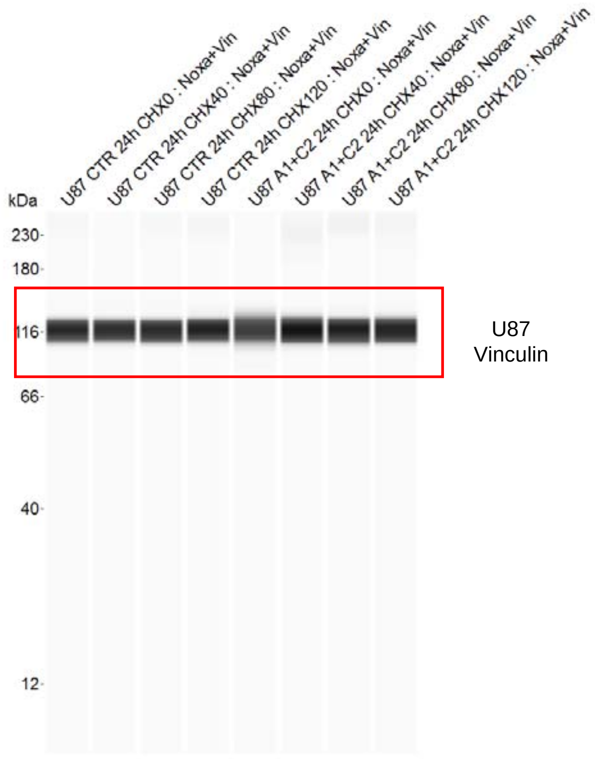
U87 CTR 24h CHX0 : Noxa+Vin
U87 CTR 24h CHX40 : Noxa+Vin
U87 CTR 24h CHX80 : Noxa+Vin
U87 CTR 24h CHX120 : Noxa+Vin
U87 A1+C2 24h CHX0 : Noxa+Vin
U87 A1+C2 24h CHX40 : Noxa+Vin
U87 A1+C2 24h CHX80 : Noxa+Vin
U87 A1+C2 24h CHX120 : Noxa+Vin
kDa
230
180
116
66
40
12
U87
Vinculin

Supplementary Figure 1C

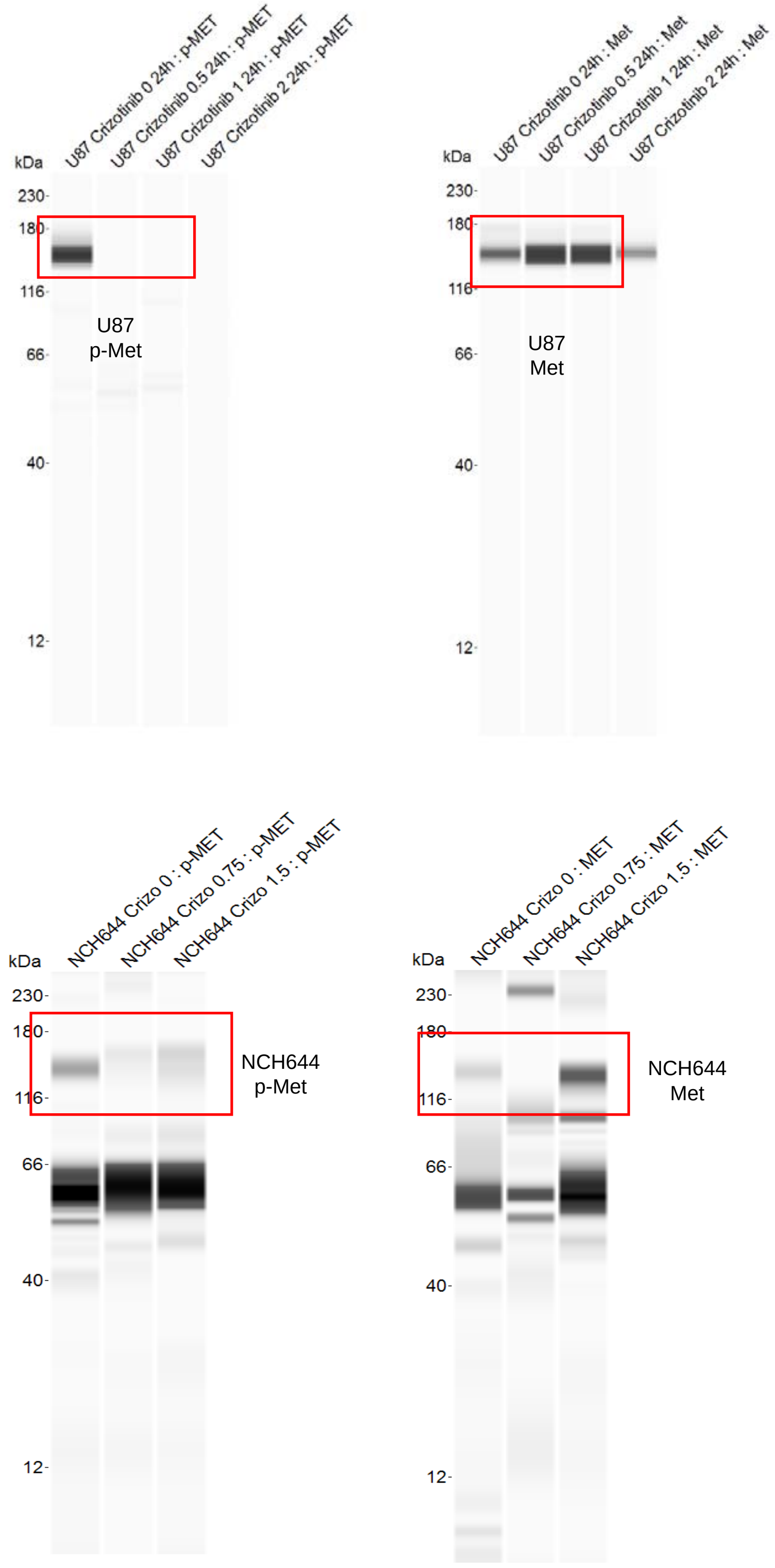

Supplementary Figure 4

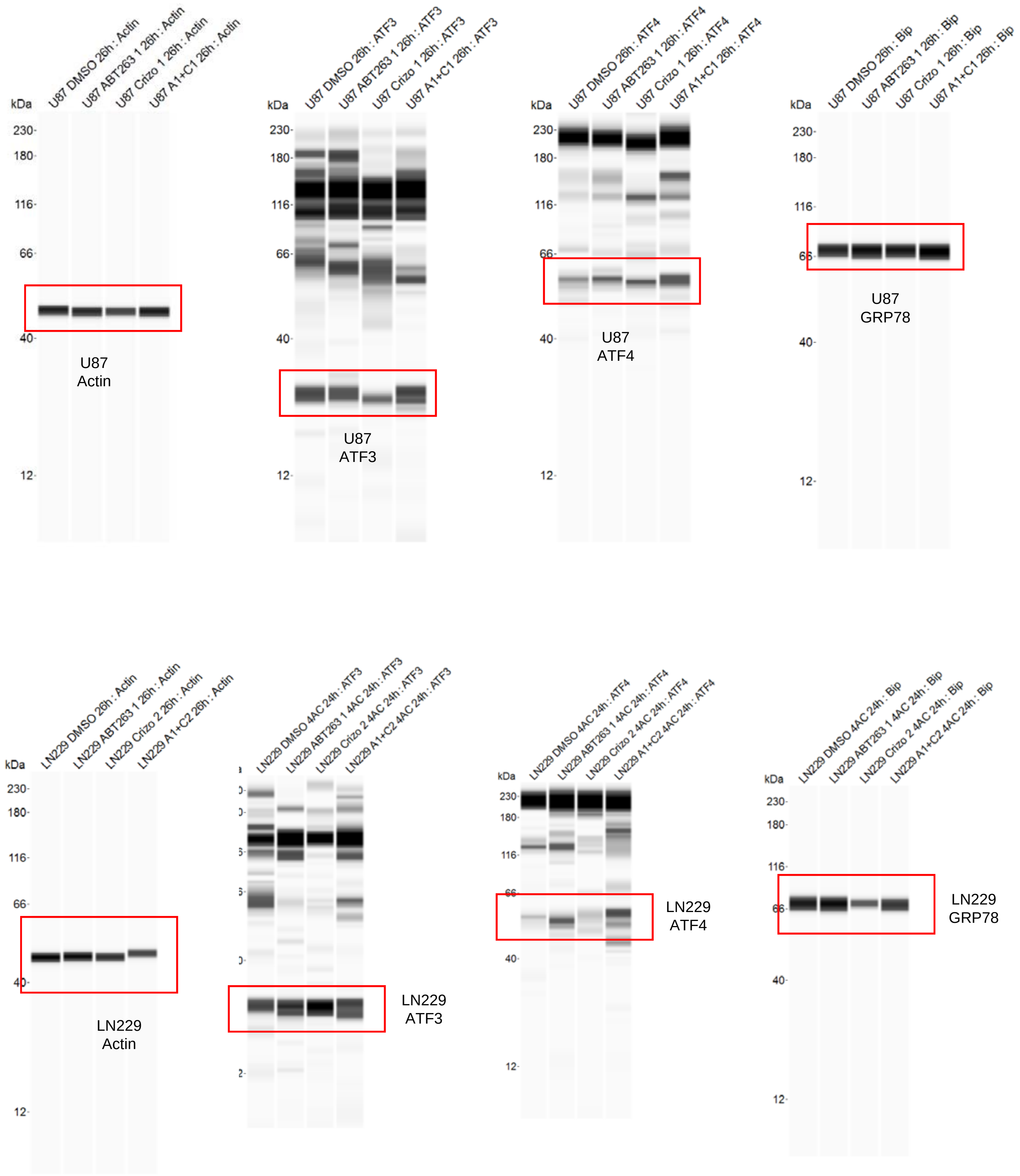

Supplement: Supplementary file 2 — Uncropped Gel Images [file 41598_2018_25802_MOESM2_ESM.pdf]
